# Security, Privacy, and Linear Function Retrieval in Combinatorial Multi-Access Coded Caching with Private Caches †

**DOI:** 10.3390/e27101033

**Published:** 2025-10-01

**Authors:** Mallikharjuna Chinnapadamala, B. Sundar Rajan

**Affiliations:** Department of Electrical Communication Engineering, IISc, Bengaluru 560012, India; chinnapadama@iisc.ac.in

**Keywords:** coded caching, linear function retrieval, security, privacy, multi-access

## Abstract

We consider combinatorial multi-access coded caching with private caches, where users are connected to two types of caches: private caches and multi-access caches. Each user has its own private cache, while multi-access caches are connected in the same way as caches are connected in a combinatorial topology. A scheme is proposed that satisfies the following three requirements simultaneously: (a) Linear Function Retrieval (LFR), (b) content security against an eavesdropper, and (c) demand privacy against a colluding set of users. It is shown that the private caches included in this work enable the proposed scheme to provide privacy against colluding users. For the same rate, our scheme requires less total memory accessed by each user and less total system memory than the existing scheme for multi-access combinatorial topology (no private caches) in the literature. We derive a cut-set lower bound and prove optimality when r≥C−1. For r<C−1, we show a constant gap of 5 under certain conditions. Finally, the proposed scheme is extended to a more general setup where different users are connected to different numbers of multi-access caches, and multiple users are connected to the same subset of multi-access caches.

## 1. Introduction

Coded caching is a promising technique to reduce network congestion during peak traffic hours by duplicating part of the contents at the users’ caches. The model consists of a single server connected to multiple users through an error-free shared link. The coded caching scheme was first proposed by Maddah-Ali and Niesen (MAN) [1], in which each user has its own dedicated/private cache. It operates in two phases: a placement phase, where each user’s cache is populated to its size, and a delivery phase, where users reveal their demands, and the server has to satisfy them. In this phase, the server exploits the content of the caches to reduce network traffic. It was shown that achieving “global caching gain” is possible by serving several users simultaneously with a single transmission. The number of files the server sends to satisfy the demands of all the users is called the rate of the system. In the MAN scheme, the subpacketization level, which refers to the number of subfiles each file is divided into, increases exponentially with the number of users. To overcome this limitation, coded caching schemes that require much lower subpacketization were proposed using Placement Delivery Arrays (PDAs) in [2].

Multi-access networks (the networks where each user has access to multiple caches) are considered in [3,4,5,6,7]. A multi-access network where each user is assigned a unique set of *r* caches out of *C* caches is introduced in [3]. For every set of *r* caches, there is a user. This network is referred to as combinatorial topology [8]. The scheme given in [3] was shown to be optimal under the assumption of uncoded placement in [8]. A more generalized setup to combinatorial topology was introduced in [8] where different users are connected to a different number r∈[0:C] of caches, and any one set of *r* caches is uniquely assigned to $K_{r}$ users, and this holds for every r∈[0:C]. A multi-access network with *K* users and *K* caches, with each user having access to *r* caches in a cyclic wrap-around way, was considered in [4,5,6,7].

The problem of scalar linear function retrieval, where users are interested in retrieving a scalar linear function of files and not just a single file, was considered in [9]. It is shown that the rate for linear function retrieval depends on the number of linearly independent functions that are demanded. When all the demanded functions are linearly independent, the rate achieved for linear function retrieval is the same as that of the MAN scheme. The concept of demand privacy, where a user should not gain any information about the index of the file demanded by another user, was considered for FR in [10,11,12]. The LFR, along with demand privacy against colluding users, i.e., any subset of users cannot obtain any information about the demands of other users, even if they exchange their cache contents, was considered in [13]. The key idea is to use privacy keys, which are formed as random linear combinations of the files. Content security for FR was studied in [14], where the system is required to protect the library content against an eavesdropper who observes the signal transmitted by the server during the delivery phase. To achieve this, security keys shared among the users are used to secure the transmitted signal from an external eavesdropper. The possibility of providing demand privacy and security simultaneously using the idea of key superposition was shown in [15]. The schemes where each user is allowed to demand an arbitrary file from the library are referred to as File Retrieval (FR) schemes [15], and the schemes where each user demands an arbitrary linear combination of the files are referred to as Linear Function Retrieval (LFR) schemes [15]. Demand for privacy for the multi-access networks where users have access to *r* caches in a cyclic wrap-around way [6] was considered in [16]. Security, privacy, and LFR for the combinatorial topology were studied in [17] using Shamir’s secret sharing scheme [18]. In this paper, we extend our previous work [19], which addressed security and privacy in the combinatorial topology with private caches.

Edge caching has emerged as an essential solution for delivering content with low latency and high efficiency in IoT (Internet of Things) networks [20]. Integrating mobile edge computing with IoT applications has been shown to reduce energy consumption and latency by offloading computation tasks to network-edge servers [21]. Edge caching helps minimize service latency and satellite bandwidth usage in applications such as hybrid satellite-IoT networks [22]. In the network model considered in this work, each user with a private cache is also connected to multiple multi-access caches, which are shared among multiple users, so that content delivery in distributed IoT environments, such as smart cities and industrial automation systems, can be done efficiently. Multi-layered cache structures are commonly employed in various IoT environments, and they are discussed in [23,24,25]. Recently, medical IoT devices and Wireless Medical Sensor Networks (WMSN) have become increasingly important. However, medical data are at high risk from malware and human interference for financial exploitation [26]. The WMSN focuses on protecting the network from unauthorized users and eavesdroppers [27]. Key management and user authentication in IoT environments were explored in [28]. The protection of medical data in Implantable Medical Devices (IMD) such as insulin pumps was discussed in [29]. In this work, we consider content security and demand privacy, which can be applied in a medical IoT environment.

**Figure 1:**
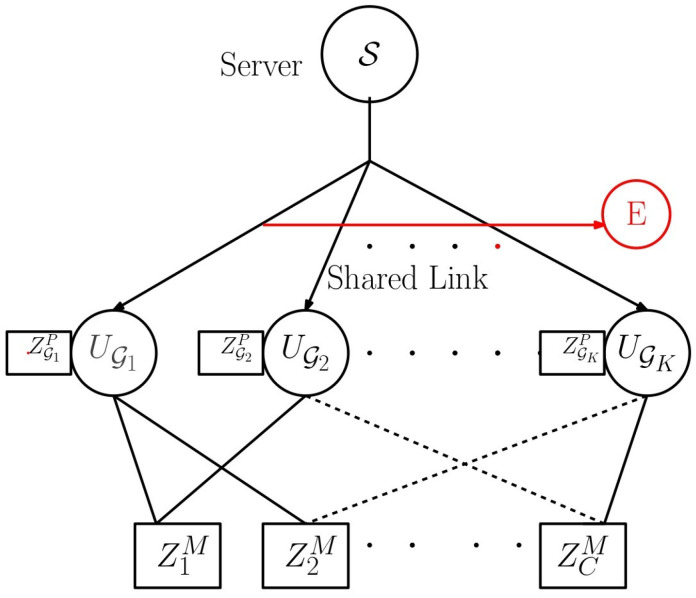
Combinatorial topology with private caches.

Our work is closely related to the information-theoretic foundations of distributed storage systems (DSS). Distributed storage systems (DSS) store data redundantly across multiple nodes to ensure reliability and efficiency [30]. In our model (Figure 1), the file library $W_{\left[N\right]}$ is stored in the server and later, during the placement phase, is distributed across the caches. In DSS, users access a subset of nodes to retrieve content, whereas in coded caching, users access a subset of the cache and retrieve content using the cache content and also the transmissions made by the server. The concept of demand privacy in our model is analogous to private information retrieval (PIR) [31,32,33], where the user retrieves data without revealing its index to the servers. The security aspect considered in this work is similar to the security in the wiretap channel II [34].

### 1.1. System Model

The system model considered in this work is shown in Figure 1. We refer to it as combinatorial topology with private caches. The model consists of a server connected to *K* users through an error-free shared link. There are two types of caches: (1) private caches and (2) multi-access caches. Each user has a private cache and also has access to a unique set of *r* out of *C* multi-access caches. For every set of *r* multi-access caches, there is a user. So, the total number of users is K=Cr. The size of each multi-access cache is $M_{M}$ files, and that of each private cache is $M_{P}$ files. An external eavesdropper *E* is assumed to observe the signals transmitted by the server.

### 1.2. Contributions and Paper Organization

We consider security, privacy, and LFR for the combinatorial topology with the private caches model shown in Figure 1. Without private caches, we have reported security, demand privacy, and LFR in [17]. The technical contributions in this work are summarized as follows:

- We propose a scheme for the combinatorial topology with private caches that provides security, privacy, and LFR.
- The scheme in [17] does not provide privacy against colluding users, but the private caches included in this work enable the proposed scheme to provide privacy against colluding users.
- It is shown that to achieve the same rate both in [17] and in the proposed scheme, the total memory accessed by each user is less in the proposed scheme.
- Next, we compare the global cache, which is the total memory size of all the caches used in the entire system. It turns out that the global cache memory requirement is also less for the system considered in this work compared to that in [17].
- We derive a lower bound on the rate using cut-set arguments, and we prove that the proposed scheme is optimal when r≥C−1.
- When r<C−1 and at $M_{M}=\frac{N}{C},M_{P}=1-\frac{r}{C}$ the proposed scheme is within a multiplicative gap of 5 from the optimal when K≤5(r+1).
- As a special case, when r=1, the proposed scheme recovers the MAN-PDA-based Secure Private LFR (SP-LFR) scheme in [15].
- The proposed scheme is extended to a more general setup where different users are connected to different numbers of multi-access caches, and multiple users are connected to the same subset of multi-access caches.
- We show numerical plots to compare the performance of the proposed scheme with the SP-LFR scheme in [17] and the MAN-PDA-based SP-LFR scheme in [15].

The paper is organized as follows: Section 2 introduces the problem setup and presents some preliminaries. A motivating example and criteria on the minimum cache size for security are provided in Section 3. Section 4 contains the proposed scheme, and the extension to a more generalized setup is presented in Section 5. Section 6 contains the main results, and a discussion of our results using numerical evaluations is presented in Section 7. Finally, Section 8 concludes the paper.

### 1.3. Notations and Information-Theoretic Preliminaries

The set {1,2,3,…n} is denoted as [n]. |X| denotes cardinality of the set X. For two non-negative integers l,m such that l≤m, we use [l:m] to denote the set {l,l+1,…m}. ⌊x⌋ denotes the largest integer less than or equal to *x*. A finite field of size *q* is represented as $F_{q}$. For a set A, we define $X_{A}:=\left\{X_{i}:i\in A\right\}$. For two sets A and B, A∖B denotes the elements in A but not in B. For two non-negative integers n,m, we have nm=n!(n−m)!m!, if n≥m, and nm=0 if n<m.

In addition to the notations, we briefly recall the main information-theoretic tools used in this work. For a random variable *X*, H(X) denotes its entropy, and for two random variables *X* and *Y*, I(X;Y) denotes their mutual information. The files in the library are assumed to be independent and uniformly distributed over $F_{2}^{F}$.

## 2. Problem Setup and Preliminaries

In this section, we introduce the problem setup and discuss some preliminaries. We begin by formally defining the problem setting, followed by a brief overview of the MAN scheme for multi-access coded caching [3] and the generalized combinatorial topology framework [8].

Recall the system model shown in Figure 1. The server S consists of a library of *N* files, $W_{\left[N\right]}=\left\{W_{1},W_{2},\ldots ,W_{N}\right\}$, each of size *F* bits. Let K denote the set of *K* users that are connected to the server through an error-free shared link. There are *C* caches of size $M_{M}$ files, which we refer to as multi-access caches, and *K* caches of size $M_{P}$ files, which we refer to as private caches. Each user is connected to a unique set of *r* caches out of *C* multi-access caches. Let the set of all *r*-sized subsets of [C] be denoted by

(1) $$\Omega_{r}≜\left\{G:G\subseteq \left[C\right],|G|=r\right\}.$$

For every set of *r* multi-access caches, there is a user. So, we have K=Cr users and these are represented as $U_{G},G\in \Omega_{r}$. Each user is also connected to a unique private cache. The system operates in two phases: the placement phase and the delivery phase. Each phase is described in the following subsections.

### 2.1. Placement Phase

During this phase, all the multi-access and private caches are filled by the server. The server privately generates a randomness *P* from some finite alphabet P and fills the private cache of each user using the cache function

(2) $$\psi_{G}:P\times {\left(F_{2}\right)}^{NF}\to {\left(F_{2}\right)}^{M_{P}F},\;G\in \Omega_{r}.$$

The cached content of the private cache for user $U_{G},G\in \Omega_{r}$, is denoted by

(3) $$Z_{G}^{P}:=\psi_{G}\left(P,W_{\left[N\right]}\right).$$

The cached content of multi-access cache c∈[C] is denoted by

(4) $$Z_{c}^{M}:=\zeta_{c}\left(W_{\left[N\right]}\right),$$

where $\zeta_{c}:{\left(F_{2}\right)}^{NF}\to {\left(F_{2}\right)}^{M_{M}F}$.

A user $U_{G}$ has access to the multi-access cache content $Z_{c}^{M}$ if c∈G. The total multi-access cache content available to user $U_{G}$ is denoted as

$$Z_{G}^{M}:=\underset{i\in G}{⋃}Z_{i}^{M}.$$

User $U_{G}$ also has access to the content of the private cache $Z_{G}^{P}$. The total cache content accessible to user $U_{G}$ is given by

(5) $$Z_{G}:=Z_{G}^{M}\cup Z_{G}^{P}.$$

### 2.2. Delivery Phase

The demand vector of a user $U_{G}$ is denoted by $d_{G}=\left(d_{G,1},\ldots ,d_{G,N}\right)\in F_{2}^{N}$. Thus, user $U_{G}$ is interested in retrieving the linear combination

(6) $$W_{d_{G}}≜d_{G,1}W_{1}⊕\ldots ⊕d_{G,N}W_{N}.$$

In order to satisfy the demands of all the users, the server transmits

(7) $$X:=\phi (P,d_{\Omega_{r}},W_{\left[N\right]}),$$

where $\phi :P\times {\left(F_{2}\right)}^{KN}\times {\left(F_{2}\right)}^{NF}\to {\left(F_{2}\right)}^{RF}$, and the quantity *R* is referred to as the rate of the system.

The file library $W_{\left[N\right]}$, the randomness *P*, and the set of demands $\left\{d_{G},\forall \;G\in \Omega_{r}\right\}$ are mutually independent. The following conditions must be satisfied by the delivery scheme:

- *Correctness*: Each user should be able to recover its demanded function:
  (8) $$H\left(W_{d_{G}}|X,d_{G},Z_{G}\right)=0,\;\forall \;G\in \Omega_{r}.$$
- *Security*: An external eavesdropper observing the server’s transmissions should learn nothing about the file library:
  (9) $$I(W_{\left[N\right]};X)=0.$$
- *Privacy*: Any set of colluding users should not know anything about the demands of other users:
  (10) $$I\left(d_{\Omega_{r}\setminus H};X,d_{H},Z_{H}\right)=0,\;\forall \;H\subset \Omega_{r},H\neq \emptyset .$$

**Definition** **1.** *We say that the triplet $\left(M_{M},M_{P},R\right)$ is achievable if there exists a scheme that satisfies the conditions in (8)–(10) with rate R and memory pair $\left(M_{M},M_{P}\right)$. The optimal rate for the given setting is defined as*

(11) $$R^{*}=\inf\left\{R:\left(M_{M},M_{P},R\right)\;is\;achievable\right\}.$$

Now, we discuss some preliminaries in the following sub-sections.

### 2.3. MAN Scheme for Multi-Access Coded Caching [3]

A multi-access network consisting of *C* caches and *K* users, where each user has access to a unique set of *r* caches, was considered in [3]. For every distinct set of *r* caches, a user is associated. This setup supports a large number of users while maintaining low subpacketization levels. The network is referred to as combinatorial topology [8], and the scheme proposed in [3] was proven to be optimal under the assumption of uncoded placement in [8]. The widely known Maddah-Ali–Niesen (MAN) scheme is obtained as a special case of the scheme in [3] when r=1.

The problem setup is as follows: A server containing *N* files, denoted by $W_{1},W_{2},\ldots ,W_{N}$, each of size *F* bits, is connected to *K* users through an error-free shared link. Each user is connected to a unique set of *r* out of the *C* caches. Each cache is of size *M* files, and the content of cache c∈[C] is denoted by $Z_{c}$.

In the placement phase, each file is divided into Ct subfiles, where $t=\frac{CM}{N}$. The $c^{\mathrm{th}}$ cache is filled as follows:

$$Z_{c}=\{W_{i,T}:c\in T,T\subset \left[C\right],|T|=t,\;\forall i\in \left[N\right]\}.$$

Let $d_{U}$ denote the demand of the user connected to the set U⊂[C] with |U|=r. For each subset S⊂[C] such that |S|=t+r, the server makes the following transmission:

$$\underset{\begin{array}{c} U\subset S\\ |U|=r \end{array}}{⨁}W_{d_{U},S\setminus U.}$$

The achieved rate is given by

R=Ct+rCt.

### 2.4. Genralized Combinatorial Topology (GCT) [8]

A more generalized multi-access setup known as Generalized Combinatorial Topology (GCT) was introduced in [8]. In this setup, every set of *r* caches out of *C* caches are connected to $K_{r}$ users for every r∈[0:C]. So, the total number of users in the system is

K=∑r=0CKrCr.

In the generalized combinatorial topology, $K_{r}$ users are connected to any one set of *r* caches. There are KrCr users that are connected to exactly *r* caches. This generalized combinatorial topology can be described using a C+1 length vector $K_{GCT}=\left(K_{0},\ldots K_{C}\right)$. For example, in GCT with C=4 caches and $K_{GCT}=\left(0,0,2,3,0\right)$, every set of 2 caches is connected to 2 users, and every set of 3 caches is connected to 3 users. No user is connected to all the 4 caches and also to only one cache. There is no user who is not connected to any cache. As a special case, when $K_{GCT}=\left(0,0,.,1,0,..0\right)$, where 1 is in the (r+1)th position, GCT recovers the combinatorial topology. For t∈[0:C], the placement for GCT is the same as that of combinatorial topology. The delivery procedure is also the same. For every r∈[0:C], the delivery procedure for the combinatorial topology given in [3] is repeated $K_{r}$ times. So, the rate achieved is

RG=∑r=0CKrCt+rCt.

## 3. Motivating Example and Minimum Cache Size Criteria for Security

In this section, we present an example and derive the minimum cache size criteria required for security.

**Example** **1.** *Consider C=3,r=2,N=3. Each file is divided into 3 subfiles. Thus, $W_{i}=\left\{W_{i,1},W_{i,2},W_{i,3}\right\}\;\forall \;i\in \left[3\right]$. Number of users, K=Cr=32=3. The 3 users are $\left\{U_{\left\{1,2\right\}},U_{\left\{1,3\right\}},U_{\left\{2,3\right\}}\right\}$. The server generates a random variable $V_{\left\{1,2,3\right\}}$ independently and uniformly from $F_{2}^{F/3}$. This is called a security key. Now, the server generates K=3 random vectors as follows:*

$$p_{G}≜{\left(p_{G,1},p_{G,2},p_{G,3}\right)}^{T}∼Unif\left\{F_{2}^{3}\right\},\forall \;G\in \Omega_{2}.$$

*Using these three random vectors, the server generates privacy keys as follows:*

$$\begin{array}{cc} W_{p_{\left\{1,2\right\}},3}= & p_{\{1,2\},1}W_{1,3}⊕p_{\{1,2\},2}W_{2,3}⊕p_{\{1,2\},3}W_{3,3},\\ W_{p_{\left\{1,3\right\}},2}= & p_{\{1,3\},1}W_{1,2}⊕p_{\{1,3\},2}W_{2,2}⊕p_{\{1,3\},3}W_{3,2},\\ W_{p_{\left\{2,3\right\}},1}= & p_{\{2,3\},1}W_{1,1}⊕p_{\{2,3\},2}W_{2,1}⊕p_{\{2,3\},3}W_{3,1}. \end{array}$$

*Let,*

$$D_{\{1,2\},3}:=V_{\left\{1,2,3\right\}}⊕W_{p_{\left\{1,2\right\}},3},\;D_{\{1,3\},2}:=V_{\left\{1,2,3\right\}}⊕W_{p_{\left\{1,3\right\}},2},\;D_{\{2,3\},1}:=V_{\left\{1,2,3\right\}}⊕W_{p_{\left\{2,3\right\}},1}.$$

*Now, let us look at the placement. The multi-access caches are filled as follows:*

$$Z_{1}^{M}=\left\{W_{1,1},W_{2,1},W_{3,1}\right\},\;Z_{2}^{M}=\left\{W_{1,2},W_{2,2},W_{3,2}\right\},\;Z_{3}^{M}=\left\{W_{1,3},W_{2,3},W_{3,3}\right\}.$$

*Based on the above placement, the size of each multi-access cache is 1. The private caches are filled as follows:*

$$Z_{\left\{1,2\right\}}^{P}=\left\{D_{\{1,2\},3}\right\},\;Z_{\left\{1,3\right\}}^{P}=\left\{D_{\{1,3\},2}\right\},\;Z_{\left\{2,3\right\}}^{P}=\left\{D_{\{2,3\},1}\right\}.$$

*Based on the above placement, the size of each private cache is 1/3. Let user $U_{\left\{1,2\right\}}$ request $W_{1}$, user $U_{\left\{1,3\right\}}$ request $W_{2}$, and user $U_{\left\{2,3\right\}}$ request $W_{3}$. To satisfy these demands, the server transmits the following:*

$$\begin{array}{cc} X= & V_{\left\{1,2,3\right\}}⊕W_{1,3}⊕W_{p_{\left\{1,2\right\}},3}⊕W_{2,2}⊕W_{p_{\left\{1,3\right\}},2}⊕W_{3,1}⊕W_{p_{\left\{2,3\right\}},1},\\ q_{G}= & p_{G}⊕d_{G}\;\forall \;G\in \Omega_{2}. \end{array}$$

*By the above transmissions, each user will be able to get their demands. Now, consider the user $U_{\left\{1,2\right\}}$. It has access to $D_{\{1,2\},3}$ from its private cache. It can get $W_{2,2}⊕W_{p_{\left\{1,3\right\}},2}=q_{\{1,3\},1}W_{1,2}⊕q_{\{1,3\},2}W_{2,2}⊕q_{\{1,3\},3}W_{3,2}$ as $q_{\left\{1,3\right\}}=\left(q_{\{1,3\},1},q_{\{1,3\},2},q_{\{1,3\},3}\right)$ is sent by the server, and $W_{1,2},W_{2,2},W_{3,2}$ are accessible to it from cache 1. Similarly, it can get $W_{3,1}⊕W_{p_{\left\{2,3\right\}},1}$. Thus, it can get the required subfile $W_{1,3}$. In the same way, the other users can also get the files they want. The transmission is protected by a security key. So, it is secure from any external eavesdropper. Since $q_{G}$ for all $G\in \Omega_{2}$ is uniformly distributed over $F_{2}^{3}$, no user can know the demands of the other users. The rate achieved is $\frac{1}{3}$. This completes Example 1.*

The security keys are stored in the caches to provide security. So, there is a minimum memory required to achieve security. Now, we show that for secure delivery, the multi-access cache memory size $M_{M}$ and the private cache memory size $M_{P}$ should satisfy the condition $CM_{M}+KM_{P}\geq K$. The cache memory sizes in Example 1 satisfy this condition.

**Proposition** **1.** *For a combinatorial topology with private caches, the system satisfies the security condition in (9) when N≥K, if the multi-access cache memory size $M_{M}$ and the private cache memory size $M_{P}$ satisfy the following inequality:*

(12) $$CM_{M}+KM_{P}\geq K.$$

**Proof.**  The detailed proof is given in Appendix A. □

**Remark** **1.** *If $M_{M}=0$, then $M_{P}$ should be at least one. This is the same condition given for the dedicated cache setup in [14]. If $M_{P}=0$, then $M_{M}$ should be at least K/C. This is the condition for the combinatorial topology.*

## 4. The Proposed Scheme

In this section, we present a scheme that satisfies the conditions in (8)–(10). The procedure is given below.

The server divides the *n* files $W_{1},W_{2},\ldots W_{N}$ in the following way for t∈[0:C−r]

(13) $$W_{i}=\left\{W_{i,T}:T\in \Omega_{t}\right\},\forall \;i\in \left[N\right].$$

For any vector $a=(a_{1},a_{2},\ldots ,a_{n})$, we define the following:

$$W_{a,T}:=\underset{n\in [N]}{⨁}a_{n}W_{n,T}\;\forall \;T\in \Omega_{t}.$$

### 4.1. Placement Phase

During this phase, the server generates privacy and security keys to provide demand privacy and content security, respectively. The number of security keys generated is Ct+r. The security keys $\left\{V_{S}:S\in \Omega_{t+r}\right\}$ are generated independently and uniformly from F2F/Ct. As there are Cr users, the server generates Cr random vectors $\left\{p_{G}:G\in \Omega_{r}\right\}$ as follows:

(14) $$p_{G}≜{\left(p_{G,1},\ldots p_{G,N}\right)}^{T}∼Unif\left\{F_{2}^{N}\right\},\forall \;G\in \Omega_{r}.$$

By using the above random vectors, CrC−rt privacy keys, denoted by $\left\{W_{p_{G},T}:G\in \Omega_{r},T\in \Omega_{t},G\cap T=\emptyset \right\}$ are generated as

(15) $$W_{p_{G},T}=\underset{n\in [N]}{⨁}p_{G,n}.W_{n,T}.$$

Let us define $D_{G,T}$ as follows:

(16) $$D_{G,T}≜W_{p_{G},T}⊕V_{G\cup T},\;G\in \Omega_{r},T\in \Omega_{t},G\cap T=\emptyset .$$

The placement of the mutli-access caches and the private caches is done as follows:

(17) $$\begin{array}{cc} Z_{c}^{M}= & \{W_{i,T}:c\in T,T\in \Omega_{t},\;\forall \;i\in \left[N\right]\}, \end{array}$$

(18) $$\begin{array}{cc} Z_{G}^{P}= & \{D_{G,T}:G\in \Omega_{r},T\in \Omega_{t},G\cap T=\emptyset \}. \end{array}$$

*Cache Memory*: Based on the above placement, the cache sizes are given as follows:

(19) MM=NtC,MP=C−rtCt.

### 4.2. Delivery Phase

During this phase, user $U_{G},G\in \Omega_{r}$ demands $W_{d_{G}}$, for some $d_{G}\in F_{2}^{N}$. In order to satisfy the demands, the server transmits $X=\left[Y_{S},q_{G}\right],\;\forall \;S\in \Omega_{t+r},G\in \Omega_{r}$, where

(20) $$q_{G}=d_{G}⊕p_{G},\;\forall \;G\in \Omega_{r},$$

(21) $$Y_{S}=V_{S}⊕\underset{\begin{array}{c} G\subset S\\ |G|=r \end{array}}{⨁}W_{q_{G},S\setminus G}\;\forall \;S\in \Omega_{t+r}.$$

*Correctness*: Each user should be able to retrieve its demanded function. Consider a transmission $Y_{S}$, $S\in \Omega_{t+r}$, and a user $U_{B}$ such that B⊂S. The transmission $Y_{S}$ can be written as

(22) $$Y_{S}=V_{S}⊕W_{d_{B},S\setminus B}⊕W_{p_{B},S\setminus B}\underset{\begin{array}{c} G\subset S\\ |G|=r\\ G\neq B \end{array}}{⨁}W_{d_{G},S\setminus G}⊕W_{p_{G},S\setminus G}.$$

The user $U_{B}$ can get $D_{B,S\setminus B}=W_{p_{B},S\setminus B}⊕V_{S}$ from the content of its private cache $Z_{B}^{p}$. Now, consider the term $W_{d_{G},S\setminus G}⊕W_{p_{G},S\setminus G}$, and it can be written in the following way

(23) $$\left(p_{G,1}⊕d_{G,1}\right)W_{1,S\setminus G}⊕\ldots ⊕\left(p_{G,1}⊕d_{G,N}\right)W_{N,S\setminus G}.$$

The coefficients of $W_{i,S\setminus G},\forall \;i\in \left[N\right]$ are known to the user as $q_{G},\forall \;G\in \Omega_{r}$ are sent by the server and the user $U_{B}$ has access to the subfiles $W_{i,S\setminus G},G\subset S,\left|G\right|=r,G\neq B\;\forall \;i\in \left[N\right]$. So, it can calculate the entire term. Thus, the user will be able to get $W_{d_{B},S\setminus B}$. Similarly, from all the transmissions corresponding to such (t+r)-sized subsets S, with B⊂S, the user $U_{B}$ gets the missing subfiles of its demand. Similarly, any user $U_{G},G\subset C,\left|G\right|=r$ can recover the missing subfiles of its demanded file.

*Privacy*: We now prove the condition for privacy given in (10). Each user $U_{G},G\in \Omega_{r}$ has access to a private cache and *r* multi-access caches. Let $Z_{H},H\subset \Omega_{r}$ denote the content of the caches of all colluding users. We have

(24a) $$\begin{array}{cc} \; & I(d_{\Omega_{r}\setminus H};X,d_{H},Z_{H}) \end{array}$$

(24b) $$\begin{array}{cc} \; & \leq I(d_{\Omega_{r}\setminus H};X,d_{H},Z_{H},W_{\left[N\right]}) \end{array}$$

(24c) $$\begin{array}{cc} \; & =I(d_{\Omega_{r}\setminus H};q_{\Omega_{r}},{\left\{Y_{S}\right\}}_{S\in \Omega_{t+r}},d_{H},Z_{H},W_{\left[N\right]}) \end{array}$$

(24d) $$\begin{array}{cc} \; & \leq I(d_{\Omega_{r}\setminus H};V_{\Omega_{t+r}},q_{\Omega_{r}},d_{H},Z_{H},W_{\left[N\right]}) \end{array}$$

(24e) $$\begin{array}{cc} \; & =0, \end{array}$$

where (24d) comes from the fact that ${\left\{Y_{S}\right\}}_{S\in \Omega_{t+r}}$ is determined by $q_{\Omega_{r}},V_{\Omega_{t+r}},W_{\left[N\right]}$ and (24e) comes from the fact that $d_{\Omega_{r}\setminus H}=q_{\Omega_{r}\setminus H}⊕p_{\Omega_{r}\setminus H}$ is independent of $V_{\Omega_{t+r}},q_{\Omega_{r}},d_{H},Z_{H},W_{\left[N\right]}$ as $p_{\Omega_{r}/H}$ is independently and uniformly distributed over $F_{2}^{N}$.

*Security*: We prove the security condition in (9) as follows.

(25a) $$\begin{array}{cc} I(W_{\left[N\right]};X) & =I(W_{\left[N\right]};q_{\Omega_{r}},{\left\{Y_{S}\right\}}_{S\in \Omega_{t+r}}) \end{array}$$

(25b) $$\begin{array}{cc} \; & =I\left(W_{\left[N\right]};q_{\Omega_{r}}\right)+I\left(W_{\left[N\right]};{\left\{Y_{S}\right\}}_{S\in \Omega_{t+r}}|q_{\Omega_{r}}\right) \end{array}$$

(25c) $$\begin{array}{cc} \; & =0, \end{array}$$

where (25c) comes from the fact that $q_{\Omega_{r}}$ is independent of $W_{\left[N\right]}$ and ${\left\{Y_{S}\right\}}_{S\in \Omega_{t+r}}$ is independent of $W_{\left[N\right]}$, $q_{\Omega_{r}}$ because the random variables $V_{\Omega_{t+r}}$ are independently and uniformly generated from F2F/Ct.

**Remark** **2** (Connection to Wiretap Channel II). *The use of independent security keys in our scheme is closely related to the coset coding technique used in the Wiretap Channel II (WTC-II) model [34]. In WTC-II, the message is embedded in a coset of a linear code, and a random vector (key) selects the specific codeword within the coset. The legitimate receiver, who observes all transmitted symbols, can uniquely identify both the coset and the message, while an eavesdropper observing fewer than d symbols (where d is the minimum distance of the dual code) cannot distinguish between different cosets, thereby learning nothing about the message. Analogously, in our scheme, each multicast transmission is masked by an independent random key stored in the private caches of legitimate users. These keys serve the same role as coset vectors in WTC-II: they ensure that an eavesdropper, even if it observes the entire broadcast, obtains no information about the library, while legitimate users can recover the desired content by combining the transmissions with the keys stored in private caches.*

**Remark** **3.** *When r=1, our setup reduces to a dedicated cache setting, and the proposed scheme coincides with the MAN-PDA-based SP-LFR scheme in [15]. Since the security condition cannot be satisfied when $M_{M}=0$ and $M_{P}=0$, the first achievable point at t=0 yields $M_{M}=0$ and $M_{P}=1$. However, if security is compromised, demand privacy alone can be ensured with $M_{M}=0$ and $M_{P}=0$ by simply transmitting all N files.*

**Example** **2.** *Consider C=5, r=3, t=2, and N=10. The number of users is K=53=10, and each file is divided into 52=10 subfiles.*

*The users are:*

$$U_{\left\{1,2,3\right\}},U_{\left\{1,2,4\right\}},U_{\left\{1,2,5\right\}},U_{\left\{1,3,4\right\}},U_{\left\{1,3,5\right\}},U_{\left\{1,4,5\right\}},U_{\left\{2,3,4\right\}},U_{\left\{2,3,5\right\}},U_{\left\{2,4,5\right\}},U_{\left\{3,4,5\right\}}.$$

*Each file $W_{i},\forall \;i\in \left[10\right]$ is divided into the following subfiles:*

$$W_{i,\{1,2\}},W_{i,\{1,3\}},W_{i,\{1,4\}},W_{i,\{1,5\}},W_{i,\{2,3\}},W_{i,\{2,4\}},W_{i,\{2,5\}},W_{i,\{3,4\}},W_{i,\{3,5\}},W_{i,\{4,5\}}.$$

***Placement Phase:** The server first generates 55=1 security key $V_{\left\{1,2,3,4,5\right\}}$ independently and uniformly from $F_{2}^{F/10}$. Then, it generates K=10 random vectors as follows:*

$$p_{G}={\left(p_{G,1},\ldots ,p_{G,10}\right)}^{T}∼\mathrm{Unif}\left\{F_{2}^{10}\right\},\;\forall \;G\in \Omega_{3}.$$

*The privacy keys, denoted by $\left\{W_{p_{G},T}:G\in \Omega_{3},T\in \Omega_{2},G\cap T=\emptyset \right\}$, are generated as*

$$W_{p_{G},T}=\underset{n\in [10]}{⨁}p_{G,n}\cdot W_{n,T}.$$

*Let,*

$$D_{G,T}=W_{p_{G},T}⊕V_{G\cup T},\;G\in \Omega_{3},T\in \Omega_{2},G\cap T=\emptyset .$$

*These are called keys. The placement step stores subfiles in multi-access caches and keys in private caches, so that security and privacy are guaranteed in the delivery phase.*

*The multi-access cache placement is done as follows:*

$$Z_{1}^{M}=\left\{W_{i,\{1,2\}},W_{i,\{1,3\}},W_{i,\{1,4\}},W_{i,\{1,5\}}\;\forall i\in \left[10\right]\right\},$$

$$Z_{2}^{M}=\left\{W_{i,\{1,2\}},W_{i,\{2,3\}},W_{i,\{2,4\}},W_{i,\{2,5\}}\;\forall i\in \left[10\right]\right\},$$

$$Z_{3}^{M}=\left\{W_{i,\{1,3\}},W_{i,\{2,3\}},W_{i,\{3,4\}},W_{i,\{3,5\}}\;\forall i\in \left[10\right]\right\},$$

$$Z_{4}^{M}=\left\{W_{i,\{1,4\}},W_{i,\{2,4\}},W_{i,\{3,4\}},W_{i,\{3,5\}}\;\forall i\in \left[10\right]\right\},$$

$$Z_{5}^{M}=\left\{W_{i,\{1,5\}},W_{i,\{2,5\}},W_{i,\{3,5\}},W_{i,\{4,5\}}\;\forall i\in \left[10\right]\right\}.$$

*The private cache placement is performed as follows:*

$$\begin{array}{cccc} Z_{\left\{1,2,3\right\}}^{P} & =\{D_{\left\{1,2,3\},\{4,5\right\}}\}, & Z_{\left\{1,2,4\right\}}^{P} & =\{D_{\left\{1,2,4\},\{3,5\right\}}\},\\ Z_{\left\{1,2,5\right\}}^{P} & =\{D_{\left\{1,2,5\},\{3,4\right\}}\}, & Z_{\left\{1,3,4\right\}}^{P} & =\{D_{\left\{1,3,4\},\{2,5\right\}}\},\\ Z_{\left\{1,3,5\right\}}^{P} & =\{D_{\left\{1,3,5\},\{2,4\right\}}\}, & Z_{\left\{1,4,5\right\}}^{P} & =\{D_{\left\{1,4,5\},\{2,3\right\}}\},\\ Z_{\left\{2,3,4\right\}}^{P} & =\{D_{\left\{2,3,4\},\{1,5\right\}}\}, & Z_{\left\{2,3,5\right\}}^{P} & =\{D_{\left\{2,3,5\},\{1,4\right\}}\},\\ Z_{\left\{2,4,5\right\}}^{P} & =\{D_{\left\{2,4,5\},\{1,3\right\}}\}, & Z_{\left\{3,4,5\right\}}^{P} & =\{D_{\left\{3,4,5\},\{1,2\right\}}\}. \end{array}$$

*The memory size of the multi-access cache is 4, and the size of the private cache is 0.1. The cache memory sizes satisfy the memory condition provided in Proposition 1.*

***Delivery Phase:*** *After obtaining the demand vectors, the server transmits*

$$X=\left[Y_{\left\{1,2,3,4,5\right\}},q_{G}\right]\;\forall \;G\in \Omega_{3}^{5},$$

*where*

$$\begin{array}{cc} Y_{\left\{1,2,3,4,5\right\}} & =V_{\left\{1,2,3,4,5\right\}}⊕W_{q_{\left\{1,2,3\right\}},\left\{4,5\right\}}⊕W_{q_{\left\{1,2,4\right\}},\left\{3,5\right\}}⊕W_{q_{\left\{1,2,5\right\}},\left\{3,4\right\}}\\ & ⊕W_{q_{\left\{1,3,4\right\}},\left\{2,5\right\}}⊕W_{q_{\left\{1,3,5\right\}},\left\{2,4\right\}}⊕W_{q_{\left\{1,4,5\right\}},\left\{2,3\right\}}\\ & ⊕W_{q_{\left\{2,3,4\right\}},\left\{1,5\right\}}⊕W_{q_{\left\{2,3,5\right\}},\left\{1,4\right\}}⊕W_{q_{\left\{2,4,5\right\}},\left\{1,3\right\}}⊕W_{q_{\left\{3,4,5\right\}},\left\{1,2\right\}}, \end{array}$$

*and*

$$q_{G}=p_{G}⊕d_{G},\;\forall \;G\in \Omega_{3}.$$

*Here, the server constructs a single coded transmission $Y_{\left\{1,2,3,4,5\right\}}$, which contains linear combinations of subfiles and a security key $V_{\left\{1,2,3,4,5\right\}}$, which ensures security. The vectors $q_{G}$ are generated using $p_{G}$ and the demand vector $d_{G}$. The linear combinations are constructed using $q_{G}$.* *By the above transmissions, each user will be able to get their demands. Consider the user $U_{\left\{1,2,3\right\}}$. The term $W_{q_{\left\{1,2,4\right\}},\left\{3,5\right\}}$ can be written as $\underset{i\in [10]}{⨁}q_{\{1,2,4\},i}W_{i,\{3,5\}}$. As the user knows $q_{\left\{1,2,4\right\}}=\left(q_{\{1,2,4\},1},\ldots q_{\{1,2,4\},10}\right)$ and has access to the 3rd cache, it can calculate the term. Similarly, the user $U_{\left\{1,2,3\right\}}$ can calculate the terms $W_{q_{G},S\setminus G}\;\forall \;G\subset \left\{1,2,3,4,5\right\},\left|G\right|=3,G\neq \left\{1,2,3\right\}$. Moreover, the user $U_{\left\{1,2,3\right\}}$ can get the key $V_{\left\{1,2,3,4,5\right\}}⊕W_{p_{\left\{1,2,3\right\}},\left\{4,5\right\}}$ from its private cache $Z_{\left\{1,2,3\right\}}^{P}$. So, the user $U_{\left\{1,2,3\right\}}$ can decode $W_{d_{\left\{1,2,3\right\}},\left\{4,5\right\}}$. Similarly, all the other users can decode the files demanded by them.*

*In summary, each user combines (i) the broadcast transmission, (ii) the subfiles stored in its accessible multi-access caches, and (iii) its key in the private cache to decode the demanded file. Security is ensured because every transmission includes the security key $V_{\left\{1,2,3,4,5\right\}}$, and demand privacy is guaranteed because the vectors $q_{G}$ are independent uniform random vectors. As there is only one broadcast transmission, the achieved rate is $\frac{1}{10}$.*

## 5. Extension to a More Generalized Setup

In this section, we extend our proposed scheme to a more generalized setup.

In the combinatorial topology introduced in [3], each user is connected to *r* unique caches out of *C* caches. For every set of *r* caches, there is one user. A generalized setup, referred to as Generalized Combinatorial Topology (GCT), was introduced in [8]. In GCT, the server having *N* files is connected to multiple users through an error-free shared link. There are *C* caches, and different users are connected to a different number r∈[0:C] of caches. Any one set of *r* caches is uniquely assigned to $K_{r}$ users, and this holds for every r∈[0:C]. So, the total number of users is K=∑r=0CKrCr. In the problem setup described in Section 2, out of *C* multi-access caches, every user is connected to *r* unique caches. For every set of *r* multi-access caches, there is a user. So, the multi-access caches are connected in the same way as the caches in the combinatorial topology. Now, we consider a more generalized setup where the multi-access caches are connected in the way caches are connected in the generalized combinatorial topology. So, there are users for every value of r∈[0:C], and for the same set of *r* multi-access caches, there are $K_{r}$ users. So, let us define the vector $K_{GCT}:=\left(K_{0},K_{1},\ldots K_{C}\right)$. In addition, every user has a private cache. So, we refer to this extended setup as GCT with private caches. The users are represented as $\{U_{r,G}^{l}:r\in \left[0:C\right],G\in \Omega_{r},l\in \left[K_{r},\right\}$. Let $d_{r,G}^{l}$ be the demand vector of the user $U_{r,G}^{l}$ and $Z_{r,G}^{l}$ be the total content accessible to the user $U_{r,G}^{l}$. The size of each multi-access cache is $M_{M}$. The size of the private cache of a user that is connected to the *r* multi-access cache, where r∈[0:C], is $M_{P}\left(r\right)$. The combinatorial topology with private caches discussed in Section 4 is obtained as a special case of the GCT with private caches by choosing $K_{r}=1$ for some r∈[0:C] and $K_{s}=0,\;\;\forall \;s\neq r$. The correctness, security, and privacy conditions for the generalized combinatorial topology with private caches are as follows:

- *Correctness*: Each user should be able to retrieve its demanded function,
  (26) $$H\left(W_{d_{r,G}^{l}}|X,d_{r,G}^{l},Z_{r,G}^{l}\right)=0,\forall \;r\in \left[0:C\right],l\in \left[K_{r}\right],G\in \Omega_{r}.$$
- *Security*: An external eavesdropper who is observing the signals sent by the server should not know anything about the content of the file library,
  (27) $$I(W_{\left[N\right]};X)=0.$$
- *Privacy*: Any set of colluding users should not know anything about the demands of the other users. Let $D=\{d_{r,G}^{l}:r\in \left[0:C\right],\;G\in \Omega_{r},\;l\in \left[K_{r}\right]\}$ represent the demand vectors of all the users, $\tilde{D}\subset D,\tilde{D}\neq \emptyset$ be the set that represents the demands of the colluding users and $\tilde{Z}$ represent the content of all the colluding users. Then,
  (28) $$I(D\setminus \tilde{D};X,\tilde{D},\tilde{Z})=0.$$

The proposed scheme in Section 4 is extended to the GCT with private caches in the following way.

For t∈[0:C], each file is divided into Ct subfiles as in (13). The server generates, for t∈[0:C], ∑r=0r=CKrCt+r security keys $\left\{V_{r,S}^{l}:r\in \left[0:C\right],l\in \left[K_{r}\right],S\in \Omega_{t+r}\right\}$ independently and uniformly from F2F/Ct. Then the server generates *K* random vectors $\left\{p_{r,G}^{l}:r\in \left[0:C\right],l\in \left[K_{r}\right],G\in \Omega_{r}\right\}$ as follows:

(29) $$p_{r,G}^{l}≜{\left(p_{r,G,1}^{l},\ldots p_{r,G,1}^{l}\right)}^{T}∼Unif\left\{F_{2}^{N}\right\},\forall \;r\in \left[0:C\right],l\in \left[K_{r}\right],G\in \Omega_{r}.$$

By using the above random vectors, the privacy keys $\left\{W_{p_{r,G}^{l},T}:r\in \left[0:C\right],l\in \left[K_{r}\right],G\in \Omega_{r},T\in \Omega_{t},G\cap T=\emptyset \right\}$ are generated as in (15). Let us define $D_{r,G,T}^{l}$ as follows:

(30) $$D_{r,G,T}^{l}≜W_{p_{r,G}^{l},T}⊕V_{r,G\cup T}^{l},\;\forall \;r\in \left[0:C\right],l\in \left[K_{r}\right],G\in \Omega_{r},T\in \Omega_{t},G\cap T=\emptyset .$$

The content of multi-access cache c∈[C] is represented as $Z_{c}^{M}$. The content of the private cache of user $U_{r,G}^{l}$ is denoted as $Z_{r,l,G}^{P}$. The placement of the caches is done as follows.

(31) $$Z_{c}^{M}=\left\{W_{i,T}:c\in T,T\in \Omega_{t}\;\forall \;i\in \left[N\right]\right\},$$

(32) $$Z_{r,l,G}^{P}=\left\{D_{r,G,T}^{l}:G\in \Omega_{r},T\in \Omega_{t},G\cap T=\emptyset \right\}.$$

*Cache Memory*: By the above placement, the sizes of the caches are as follows:

(33) MM=NtC,MP(r)=C−rtCt.

The size of a user’s private cache depends on *r*, the total number of multi-access caches accessible to the user, as the number of required keys depends on *r*. During the delivery phase, the users reveal their demand vectors. Let $d_{r,G}^{l}$ be the demand vector of the user $U_{r,G}^{l}$ where $r\in \left[0:C\right],l\in \left[K_{r}\right],G\in \Omega_{r}$. So, in order to satisfy the demands, the server transmits $\forall \;r\in \left[0:C\right],l\in \left[K_{r}\right],G\in \Omega_{r},S\in \Omega_{t+r}$

(34) $$q_{r,G}^{l}=d_{r,G}^{l}⊕p_{r,G}^{l},$$

(35) $$Y_{r,S}^{l}=V_{r,S}^{l}⊕\underset{\begin{array}{c} G\subset S\\ |G|=r \end{array}}{⨁}W_{q_{r,G}^{l},S\setminus G}.$$

The transmissions given above are essentially the same transmissions made by the server in the scheme given in Section 4 repeated for every $r\in \left[0:C\right],l\in \left[K_{r}\right]$. Consider any user connected to j∈[0:C] caches and let that user be the $i^{th}$ user connected to *j* caches. So, the user is $U_{j,H}^{i},H\in \Omega_{j}$. Thus, by using the transmissions $Y_{j,E}^{i},E\in \Omega_{t+j},H\subset E$, the user can reconstruct its demanded file. The security condition is satisfied, as each transmission is protected with a security key. The demand privacy condition is also satisfied as the vectors $q_{r,G}^{l},r\in \left[0:C\right],l\in \left[K_{r}\right],G\in \Omega_{t+r}$ are independently and uniformly distributed over $F_{2}^{N}$. So, correctness, security, and demand for privacy are guaranteed. The rate achieved is RG=∑r=0CKrCt+rCt.

**Example** **3.** *Consider C=5, $K_{\mathrm{GCT}}=\left(0,0,0,2,0,0\right)$, t=2, and N=20. The total number of users is K=2Cr=253=20, and each file is divided into Ct=52=10 subfiles.*

*The 20 users are denoted as*

$$U_{3,\{1,2,3\}}^{l},U_{3,\{1,2,4\}}^{l},U_{3,\{1,2,5\}}^{l},U_{3,\{1,3,4\}}^{l},U_{3,\{1,3,5\}}^{l},$$

$$U_{3,\{1,4,5\}}^{l},U_{3,\{2,3,4\}}^{l},U_{3,\{2,3,5\}}^{l},U_{3,\{2,4,5\}}^{l},U_{3,\{3,4,5\}}^{l},\;\forall \;l\in \left[2\right].$$

*Each file $W_{i},\forall \;i\;\in \left[20\right]$ is split into the following subfiles:*

$$W_{i,\{1,2\}},W_{i,\{1,3\}},W_{i,\{1,4\}},W_{i,\{1,5\}},W_{i,\{2,3\}},W_{i,\{2,4\}},W_{i,\{2,5\}},W_{i,\{3,4\}},W_{i,\{3,5\}},W_{i,\{4,5\}}.$$

*The server generates 255=2 security keys $V_{3,\{1,2,3,4,5\}}^{1}$ and $V_{3,\{1,2,3,4,5\}}^{2}$, independently and uniformly from $F_{2}^{F/10}$. Then, it generates K=20 random vectors as follows:*

(36) $$\begin{array}{c} p_{3,G}^{l}={\left(p_{3,G,1}^{l},\ldots ,p_{3,G,20}^{l}\right)}^{T}∼\mathrm{Unif}\left(F_{2}^{20}\right),\;\forall \;l\in \left[2\right],\;G\in \Omega_{3}. \end{array}$$

*The privacy keys are generated as follows:*

(37) $$W_{p_{3,G}^{l},T}=\overset{20}{\underset{n=1}{⨁}}p_{3,G,n}^{l}\cdot W_{n,T},\;\forall \;l\in \left[2\right],\;G\in \Omega_{3},\;T\in \Omega_{2},\;G\cap T=\emptyset .$$

*Let,*

(38) $$D_{3,G,T}^{l}=W_{p_{3,G}^{l},T}⊕V_{3,G\cup T}^{l},\;\forall \;l\in \left[2\right],\;G\in \Omega_{3},\;T\in \Omega_{2},\;G\cap T=\emptyset .$$

*The placement for multi-access cache is done as follows:*

$$\begin{array}{cc} Z_{1}^{M} & =\{W_{i,\{1,2\}},W_{i,\{1,3\}},W_{i,\{1,4\}},W_{i,\{1,5\}}\;\forall i\in \left[20\right]\},\\ Z_{2}^{M} & =\{W_{i,\{1,2\}},W_{i,\{2,3\}},W_{i,\{2,4\}},W_{i,\{2,5\}}\;\forall i\in \left[20\right]\},\\ Z_{3}^{M} & =\{W_{i,\{1,3\}},W_{i,\{2,3\}},W_{i,\{3,4\}},W_{i,\{3,5\}}\;\forall i\in \left[20\right]\},\\ Z_{4}^{M} & =\{W_{i,\{1,4\}},W_{i,\{2,4\}},W_{i,\{3,4\}},W_{i,\{3,5\}}\;\forall i\in \left[20\right]\},\\ Z_{5}^{M} & =\{W_{i,\{1,5\}},W_{i,\{2,5\}},W_{i,\{3,5\}},W_{i,\{4,5\}}\;\forall i\in \left[20\right]\}. \end{array}$$

*The placement for the private caches is done as follows:*

$$\begin{array}{cccccc} Z_{3,1,\{1,2,3\}}^{P} & =\{D_{3,\{1,2,3\},\{4,5\}}^{1}\}, & Z_{3,2,\{1,2,3\}}^{P} & =\{D_{3,\{1,2,3\},\{4,5\}}^{2}\}, & Z_{3,1,\{1,2,4\}}^{P} & =\{D_{3,\{1,2,4\},\{3,5\}}^{1}\},\\ Z_{3,2,\{1,2,4\}}^{P} & =\{D_{3,\{1,2,4\},\{3,5\}}^{2}\}, & Z_{3,1,\{1,2,5\}}^{P} & =\{D_{3,\{1,2,5\},\{3,4\}}^{1}\}, & Z_{3,2,\{1,2,5\}}^{P} & =\{D_{3,\{1,2,5\},\{3,4\}}^{2}\},\\ Z_{3,1,\{1,3,4\}}^{P} & =\{D_{3,\{1,3,4\},\{2,5\}}^{1}\}, & Z_{3,2,\{1,3,4\}}^{P} & =\{D_{3,\{1,3,4\},\{2,5\}}^{2}\}, & Z_{3,1,\{1,3,5\}}^{P} & =\{D_{3,\{1,3,5\},\{2,4\}}^{1}\},\\ Z_{3,2,\{1,3,5\}}^{P} & =\{D_{3,\{1,3,5\},\{2,4\}}^{2}\}, & Z_{3,1,\{1,4,5\}}^{P} & =\{D_{3,\{1,4,5\},\{2,3\}}^{1}\}, & Z_{3,2,\{1,4,5\}}^{P} & =\{D_{3,\{1,4,5\},\{2,3\}}^{2}\},\\ Z_{3,1,\{2,3,4\}}^{P} & =\{D_{3,\{2,3,4\},\{1,5\}}^{1}\}, & Z_{3,2,\{2,3,4\}}^{P} & =\{D_{3,\{2,3,4\},\{1,5\}}^{2}\}, & Z_{3,1,\{2,3,5\}}^{P} & =\{D_{3,\{2,3,5\},\{1,4\}}^{1}\},\\ Z_{3,2,\{2,3,5\}}^{P} & =\{D_{3,\{2,3,5\},\{1,4\}}^{2}\}, & Z_{3,1,\{2,4,5\}}^{P} & =\{D_{3,\{2,4,5\},\{1,3\}}^{1}\}, & Z_{3,2,\{2,4,5\}}^{P} & =\{D_{3,\{2,4,5\},\{1,3\}}^{2}\},\\ Z_{3,1,\{3,4,5\}}^{P} & =\{D_{3,\{3,4,5\},\{1,2\}}^{1}\}, & Z_{3,2,\{3,4,5\}}^{P} & =\{D_{3,\{3,4,5\},\{1,2\}}^{2}\}. & \; \end{array}$$

*The memory size of each multi-access cache is 8, and the size of each private cache is 0.1. After receiving the demand vectors, the server transmits $X=[Y_{3,\{1,2,3,4,5\}}^{l},q_{3,G}^{l}]$, $\;\forall \;l\in \left[2\right],G\in \Omega_{3}$ where $Y_{3,\{1,2,3,4,5\}}^{1}=V_{3,\{1,2,3,4,5\}}^{1}⊕W_{q_{3,\left\{1,2,3\right\}}^{1},\left\{4,5\right\}}⊕W_{q_{3,\left\{1,2,4\right\}}^{1},\left\{3,5\right\}}⊕W_{q_{3,\left\{1,2,5\right\}}^{1},\left\{3,4\right\}}⊕W_{q_{3,\left\{1,3,4\right\}}^{1},\left\{2,5\right\}}⊕W_{q_{3,\left\{1,3,5\right\}}^{1},\left\{2,4\right\}}⊕W_{q_{3,\left\{1,4,5\right\}}^{1},\left\{2,3\right\}}⊕W_{q_{3,\left\{2,3,4\right\}}^{1},\left\{1,5\right\}}⊕W_{q_{3,\left\{2,3,5\right\}}^{1},\left\{1,4\right\}}⊕W_{q_{3,\left\{2,4,5\right\}}^{1},\left\{1,3\right\}}⊕W_{q_{3,\left\{3,4,5\right\}}^{1},\left\{1,2\right\}}$,*

*$Y_{3,\{1,2,3,4,5\}}^{2}=V_{3,\{1,2,3,4,5\}}^{2}⊕W_{q_{3,\left\{1,2,3\right\}}^{2},\left\{4,5\right\}}⊕W_{q_{3,\left\{1,2,4\right\}}^{2},\left\{3,5\right\}}⊕W_{q_{3,\left\{1,2,5\right\}}^{2},\left\{3,4\right\}}⊕W_{q_{3,\left\{1,3,4\right\}}^{2},\left\{2,5\right\}}⊕W_{q_{3,\left\{1,3,5\right\}}^{2},\left\{2,4\right\}}⊕W_{q_{3,\left\{1,4,5\right\}}^{2},\left\{2,3\right\}}⊕W_{q_{3,\left\{2,3,4\right\}}^{2},\left\{1,5\right\}}⊕W_{q_{3,\left\{2,3,5\right\}}^{2},\left\{1,4\right\}}⊕W_{q_{3,\left\{2,4,5\right\}}^{2},\left\{1,3\right\}}⊕W_{q_{3,\left\{3,4,5\right\}}^{2},\left\{1,2\right\}}$,*

*$q_{3,G}^{l}=p_{3,G}^{l}⊕d_{3,G}^{l},\;\forall \;l\in \left[2\right],G\in \Omega_{3}$.*

*By the above transmissions, each user will be able to get their demands. Consider the user $U_{3,\{1,2,3\}}^{1}$ and the transmission $Y_{3,\{1,2,3,4,5\}}^{1}$. The term $W_{q_{3,\left\{1,2,4\right\}}^{1},\left\{3,5\right\}}$ can be written as $\underset{i\in [20]}{⨁}q_{3,\{1,2,4\},i}^{1}W_{i,\{3,5\}}$. As the user knows $q_{3,\{1,2,4\}}^{1}=\left(q_{3,\{1,2,4\},1}^{1},\ldots q_{3,\{1,2,4\},20}^{1}\right)$ and has access to the 3rd cache, it can calculate the term. Similarly, the user $U_{3,\{1,2,3\}}^{1}$ can calculate the terms $W_{q_{3,G}^{1},S\setminus G}\forall G\subset \left\{1,2,3,4,5\right\},\left|G\right|=3,G\neq \left\{1,2,3\right\}$. Moreover, the user $U_{3,\{1,2,3\}}^{1}$ can get the key $V_{3,\{1,2,3,4,5\}}^{1}⊕W_{p_{3,\{1,2,3\}}^{1},\left\{4,5\right\}}$ from its private cache $Z_{3,1,\{1,2,3\}}^{P}$. So, the user $U_{3,\{1,2,3\}}^{1}$ can decode $W_{d_{3,\left\{1,2,3\right\}}^{1},\left\{4,5\right\}}$. Using the same procedure, the user $U_{3,\{1,2,3\}}^{2}$ can decode $W_{d_{3,\left\{1,2,3\right\}}^{2},\left\{4,5\right\}}$ from $Y_{3,\{1,2,3,4,5\}}^{2}$. Similarly, all the other users that are connected to the 3 multi-access caches can decode the files demanded by them. Now, let us see the demand privacy constraint. The server sends the vectors $q_{3,G}^{l},\forall \;l\in \left[2\right],G\in \Omega_{3}$ to all the users. Since the vectors are uniformly distributed, it is impossible to know anything about the demand vectors $d_{3,G}^{l},\forall \;l\in \left[2\right],G\in \Omega_{3}$. Thus, privacy is guaranteed because each user does not know the key of any other user, and the vectors $q_{3,G}^{l},\forall \;l\in \left[2\right],G\in \Omega_{3}$ are random vectors independently and uniformly distributed over $F_{2}^{20}$. Now, let us look into the aspect of security. Each signal sent by the server is protected by a security key. Therefore, an eavesdropper who continuously observes the signals sent by the server learns nothing about the file library. Since there are two transmissions, the achieved rate is $\frac{2}{10}$.*

**Example** **4.** *Consider C=5, $K_{GCT}=\left(0,0,1,1,0,0\right)$, t=2, N=20. The total number of users, K=C2+C3=53+52=20 and each file is divided in to Ct=52=10 subfiles. Ten users are connected to 2 multi-access caches, and the other ten users are connected to 3 multi-access caches. The ten users that are connected to 3 multi-access caches are represented as*

$$U_{3,\{1,2,3\}}^{1},U_{3,\{1,2,4\}}^{1},U_{3,\{1,2,5\}}^{1},U_{3,\{1,3,4\}}^{1},U_{3,\{1,3,5\}}^{1},$$

$$U_{3,\{1,4,5\}}^{1},U_{3,\{2,3,4\}}^{1},U_{3,\{2,3,5\}}^{1},U_{3,\{2,4,5\}}^{1},U_{3,\{3,4,5\}}^{1}.$$

*The other ten users that are connected to 2 multi-access caches are represented as*

$$U_{2,\{1,2\}}^{1},U_{2,\{1,3\}}^{1},U_{2,\{1,4\}}^{1},U_{2,\{1,5\}}^{1},U_{2,\{2,3\}}^{1},U_{2,\{2,4\}}^{1},U_{2,\{2,5\}}^{1},U_{2,\{3,4\}}^{1},U_{2,\{3,5\}}^{1},U_{2,\{4,5\}}^{1}.$$

*The subfiles of $W_{i},\forall \;i\in \left[20\right]$ are*

$$W_{i,\{1,2\}},W_{i,\{1,3\}},W_{i,\{1,4\}},W_{i,\{1,5\}},W_{i,\{2,3\}},W_{i,\{2,4\}},W_{i,\{2,5\}},W_{i,\{3,4\}},W_{i,\{3,5\}},W_{i,\{4,5\}}.$$

*The server generates 53+54=1+5=6 security keys $\left\{V_{r,S}^{1}:r\in \left\{2,3\right\},S\in \Omega_{2+r}\right\}$ independently and uniformly from $F_{2}^{F/10}$. Then, the server generates K=20 random vectors as follows:*

$$p_{r,G}^{1}={\left(p_{r,G,1}^{1},\ldots ,p_{r,G,20}^{1}\right)}^{T}∼\mathrm{Unif}\left(F_{2}^{10}\right),\forall \;r\in \left\{2,3\right\},\;G\in \Omega_{r}.$$

*The privacy keys, denoted by*

$$\{W_{p_{r,G}^{1},T}:r\in \left\{2,3\right\},\;G\in \Omega_{r},\;T\in \Omega_{2},\;G\cap T=\emptyset \},$$

*are generated as in (15). Let,*

$$D_{r,G,T}^{1}=W_{p_{r,G}^{1},T}⊕V_{r,G\cup T}^{1},\mathrm{for}\;r\in \left\{2,3\right\},\;G\in \Omega_{r},\;T\in \Omega_{2},\;G\cap T=\emptyset .$$

*The placement for the multi-access cache is done as follows:*

$$\begin{array}{cc} Z_{1}^{M} & =\{W_{i,\{1,2\}},W_{i,\{1,3\}},W_{i,\{1,4\}},W_{i,\{1,5\}}\;\forall \;i\in \left[20\right]\},\\ Z_{2}^{M} & =\{W_{i,\{1,2\}},W_{i,\{2,3\}},W_{i,\{2,4\}},W_{i,\{2,5\}}\;\forall \;i\in \left[20\right]\},\\ Z_{3}^{M} & =\{W_{i,\{1,3\}},W_{i,\{2,3\}},W_{i,\{3,4\}},W_{i,\{3,5\}}\;\forall \;i\in \left[20\right]\},\\ Z_{4}^{M} & =\{W_{i,\{1,4\}},W_{i,\{2,4\}},W_{i,\{3,4\}},W_{i,\{4,5\}}\;\forall \;i\in \left[20\right]\},\\ Z_{5}^{M} & =\{W_{i,\{1,5\}},W_{i,\{2,5\}},W_{i,\{3,5\}},W_{i,\{4,5\}}\;\forall \;i\in \left[20\right]\}. \end{array}$$

*The placement for the private caches is done as follows:*

$$\begin{array}{cc} \; & Z_{3,1,\{1,2,3\}}^{P}=\left\{D_{3,\{1,2,3\},\{4,5\}}^{1}\right\},\;Z_{2,1,\{1,2\}}^{P}=\left\{D_{2,\{1,2\},\{3,4\}}^{1},D_{2,\{1,2\},\{3,5\}}^{1},D_{2,\{1,2\},\{4,5\}}^{1}\right\},\\ \; & Z_{3,1,\{1,2,4\}}^{P}=\left\{D_{3,\{1,2,4\},\{3,5\}}^{1}\right\},\;Z_{2,1,\{1,3\}}^{P}=\left\{D_{2,\{1,3\},\{2,4\}}^{1},D_{2,\{1,2\},\{2,5\}}^{1},D_{2,\{1,2\},\{4,5\}}^{1}\right\},\\ \; & Z_{3,1,\{1,2,5\}}^{P}=\left\{D_{3,\{1,2,5\},\{3,4\}}^{1}\right\},\;Z_{2,1,\{1,4\}}^{P}=\left\{D_{2,\{1,4\},\{2,3\}}^{1},D_{2,\{1,4\},\{2,5\}}^{1},D_{2,\{1,4\},\{3,5\}}^{1}\right\},\\ \; & Z_{3,1,\{1,3,4\}}^{P}=\left\{D_{3,\{1,3,4\},\{2,5\}}^{1}\right\},\;Z_{2,1,\{1,5\}}^{P}=\left\{D_{2,\{1,5\},\{2,4\}}^{1},D_{2,\{1,5\},\{2,3\}}^{1},D_{2,\{1,5\},\{3,4\}}^{1}\right\},\\ \; & Z_{3,1,\{1,3,5\}}^{P}=\left\{D_{3,\{1,3,5\},\{2,4\}}^{1}\right\},\;Z_{2,1,\{2,3\}}^{P}=\left\{D_{2,\{2,3\},\{1,4\}}^{1},D_{2,\{2,3\},\{1,5\}}^{1},D_{2,\{2,3\},\{4,5\}}^{1}\right\},\\ \; & Z_{3,1,\{1,4,5\}}^{P}=\left\{D_{3,\{1,4,5\},\{2,3\}}^{1}\right\},\;Z_{2,1,\{2,4\}}^{P}=\left\{D_{2,\{2,4\},\{1,3\}}^{1},D_{2,\{2,4\},\{1,5\}}^{1},D_{2,\{2,4\},\{3,5\}}^{1}\right\},\\ \; & Z_{3,1,\{2,3,4\}}^{P}=\left\{D_{3,\{2,3,4\},\{1,5\}}^{1}\right\},\;Z_{2,1,\{2,5\}}^{P}=\left\{D_{2,\{2,5\},\{1,3\}}^{1},D_{2,\{2,5\},\{1,4\}}^{1},D_{2,\{2,5\},\{3,4\}}^{1}\right\},\\ \; & Z_{3,1,\{2,3,5\}}^{P}=\left\{D_{3,\{2,3,5\},\{1,4\}}^{1}\right\},\;Z_{2,1,\{3,4\}}^{P}=\left\{D_{2,\{3,4\},\{1,2\}}^{1},D_{2,\{3,4\},\{1,5\}}^{1},D_{2,\{3,4\},\{2,5\}}^{1}\right\},\\ \; & Z_{3,1,\{2,4,5\}}^{P}=\left\{D_{3,\{2,4,5\},\{1,3\}}^{1}\right\},\;Z_{2,1,\{3,5\}}^{P}=\left\{D_{2,\{3,5\},\{1,2\}}^{1},D_{2,\{3,5\},\{1,4\}}^{1},D_{2,\{3,5\},\{2,4\}}^{1}\right\},\\ \; & Z_{3,1,\{3,4,5\}}^{P}=\left\{D_{3,\{3,4,5\},\{1,2\}}^{1}\right\},\;Z_{2,1,\{4,5\}}^{P}=\left\{D_{2,\{4,5\},\{1,2\}}^{1},D_{2,\{4,5\},\{1,3\}}^{1},D_{2,\{4,5\},\{2,3\}}^{1}\right\}. \end{array}$$

*The memory size of each multi-access cache is 8, and the sizes of the private caches are $M^{P}\left(2\right)=0.3$ and $M^{P}\left(3\right)=0.1$. After receiving the demand vectors, the server transmits $X=[Y_{r,\Omega_{2+r}}^{1},q_{r,G}^{1}]$, $\;\forall \;r\in \left\{2,3\right\},G\in \Omega_{r}$ where*

*$Y_{3,\{1,2,3,4,5\}}^{1}=V_{3,\{1,2,3,4,5\}}^{1}⊕W_{q_{3,\left\{1,2,3\right\}}^{1},\left\{4,5\right\}}⊕W_{q_{3,\left\{1,2,4\right\}}^{1},\left\{3,5\right\}}⊕W_{q_{3,\left\{1,2,5\right\}}^{1},\left\{3,4\right\}}⊕W_{q_{3,\left\{1,3,4\right\}}^{1},\left\{2,5\right\}}⊕W_{q_{3,\left\{1,3,5\right\}}^{1},\left\{2,4\right\}}⊕W_{q_{3,\left\{1,4,5\right\}}^{1},\left\{2,3\right\}}⊕W_{q_{3,\left\{2,3,4\right\}}^{1},\left\{1,5\right\}}⊕W_{q_{3,\left\{2,3,5\right\}}^{1},\left\{1,4\right\}}⊕W_{q_{3,\left\{2,4,5\right\}}^{1},\left\{1,3\right\}}⊕W_{q_{3,\left\{3,4,5\right\}}^{1},\left\{1,2\right\}}$,*

*$Y_{2,\{1,2,3,4\}}^{1}=V_{2,\{1,2,3,4\}}^{1}⊕W_{q_{2,\left\{1,2\right\}}^{1},\left\{3,4\right\}}⊕W_{q_{2,\left\{1,3\right\}}^{1},\left\{2,4\right\}}⊕W_{q_{2,\left\{1,4\right\}}^{1},\left\{2,3\right\}}⊕W_{q_{2,\left\{2,3\right\}}^{1},\left\{1,4\right\}}⊕W_{q_{2,\left\{2,4\right\}}^{1},\left\{1,3\right\}}⊕W_{q_{2,\left\{3,4\right\}}^{1},\left\{1,2\right\}}$,*

*$Y_{2,\{1,2,3,5\}}^{1}=V_{2,\{1,2,3,5\}}^{1}⊕W_{q_{2,\left\{1,2\right\}}^{1},\left\{3,5\right\}}⊕W_{q_{2,\left\{1,3\right\}}^{1},\left\{2,5\right\}}⊕W_{q_{2,\left\{1,5\right\}}^{1},\left\{2,3\right\}}⊕W_{q_{2,\left\{2,3\right\}}^{1},\left\{1,5\right\}}⊕W_{q_{2,\left\{2,5\right\}}^{1},\left\{1,3\right\}}⊕W_{q_{2,\left\{3,5\right\}}^{1},\left\{1,2\right\}}$,*

*$Y_{2,\{1,2,4,5\}}^{1}=V_{2,\{1,2,4,5\}}^{1}⊕W_{q_{2,\left\{1,2\right\}}^{1},\left\{4,5\right\}}⊕W_{q_{2,\left\{1,4\right\}}^{1},\left\{2,5\right\}}⊕W_{q_{2,\left\{1,5\right\}}^{1},\left\{2,4\right\}}⊕W_{q_{2,\left\{2,4\right\}}^{1},\left\{1,5\right\}}⊕W_{q_{2,\left\{2,5\right\}}^{1},\left\{1,4\right\}}⊕W_{q_{2,\left\{4,5\right\}}^{1},\left\{1,2\right\}}$,*

*$Y_{2,\{1,3,4,5\}}^{1}=V_{2,\{1,3,4,5\}}^{1}⊕W_{q_{2,\left\{1,3\right\}}^{1},\left\{4,5\right\}}⊕W_{q_{2,\left\{1,4\right\}}^{1},\left\{3,5\right\}}⊕W_{q_{2,\left\{1,5\right\}}^{1},\left\{3,4\right\}}⊕W_{q_{2,\left\{3,4\right\}}^{1},\left\{1,5\right\}}⊕W_{q_{2,\left\{3,5\right\}}^{1},\left\{1,4\right\}}⊕W_{q_{2,\left\{4,5\right\}}^{1},\left\{1,3\right\}}$,*

*$Y_{2,\{2,3,4,5\}}^{1}=V_{2,\{2,3,4,5\}}^{1}⊕W_{q_{2,\left\{2,3\right\}}^{1},\left\{4,5\right\}}⊕W_{q_{2,\left\{2,4\right\}}^{1},\left\{3,5\right\}}⊕W_{q_{2,\left\{2,5\right\}}^{1},\left\{3,4\right\}}⊕W_{q_{2,\left\{3,4\right\}}^{1},\left\{2,5\right\}}⊕W_{q_{2,\left\{3,5\right\}}^{1},\left\{2,4\right\}}⊕W_{q_{2,\left\{4,5\right\}}^{1},\left\{2,3\right\}}$,*

*$q_{r,G}^{1}=p_{r,G}^{1}⊕d_{r,G}^{1},\;\forall \;r\in \left\{2,3\right\},G\in \Omega_{r}$.*

*By the above transmissions, each user will be able to get their demands. Consider the user $U_{3,\{1,2,3\}}^{1}$ and the transmission $Y_{3,\{1,2,3,4,5\}}^{1}$. The term $W_{q_{3,\left\{1,2,4\right\}}^{1},\left\{3,5\right\}}$ can be written as $\underset{i\in [20]}{⨁}q_{3,\{1,2,4\},i}^{1}W_{i,\{3,5\}}$. As the user knows $q_{3,\{1,2,4\}}^{1}=\left(q_{3,\{1,2,4\},1}^{1},\ldots q_{3,\{1,2,4\},20}^{1}\right)$ and has access to the 3rd cache, it can calculate the term. Similarly, the user $U_{3,\{1,2,3\}}^{1}$ can calculate the terms $W_{q_{3,G}^{1},S\setminus G}\forall \;G\subset \left\{1,2,3,4,5\right\},\left|G\right|=3,G\neq \left\{1,2,3\right\}$. Moreover, the user $U_{3,\{1,2,3\}}^{1}$ can get the key $V_{3,\{1,2,3,4,5\}}^{1}⊕W_{p_{3,\{1,2,3\}}^{1},\left\{4,5\right\}}$ from its private cache $Z_{3,1,\{1,2,3\}}^{P}$. So, the user $U_{3,\{1,2,3\}}^{1}$ can decode $W_{d_{3,\left\{1,2,3\right\}}^{1},\left\{4,5\right\}}$. Similarly, all the other users can decode the files demanded by them. The users that are connected to 2 caches make use of the transmissions done for every 4-sized subset of [5]. Now, let us see the demand privacy constraint. The server sends the vectors $q_{r,G}^{1},\forall \;r\in \left\{2,3\right\},G\in \Omega_{r}$ to all the users. Since the vectors are uniformly distributed, it is impossible to know anything about the demand vectors $d_{r,G}^{1},\forall \;r\in \left\{2,3\right\},G\in \Omega_{r}$. So, demand privacy is guaranteed since each user does not know the key of the other user, and the vectors $q_{r,G}^{1},\forall \;r\in \left\{2,3\right\},G\in \Omega_{r}$ are random vectors independently and uniformly distributed over $F_{2}^{20}$. Now, let us look into the aspect of security. Each signal sent by the server is protected by a security key. Therefore, an eavesdropper who continuously observes the signals sent by the server learns nothing about the file library. As there are six transmissions, the rate achieved is $\frac{6}{10}$.*

## 6. Main Results

In this section, we present the main results of this paper.

**Theorem** **1.** *Consider the combinatorial topology with private caches. For t∈[0:C−r], with $M_{M}=Nt/C$ and MP=C−rt/Ct, there exists a scheme that satisfies the conditions in (8)–(10) with the rate*

R=Ct+rCt.

**Proof.**  Consider the proposed scheme in Section 4. Each file is divided into Ct subfiles. So, the size of each subfile is 1Ct. Each transmission also has a size of 1Ct. There is a transmission for every (t+r)-sized subset of [C]. Since the lengths of $q_{G},\forall \;G\in \Omega_{r}$ are negligible compared to the file size, the total rate R=Ct+rCt. The scheme is given for integer values of *t*. For the other values of *t*, memory sharing can be used to get the rate. □

### Comparison with [17]

In this subsection, we compare the proposed scheme with the SP-LFR scheme in [17]. Security, privacy, and LFR were considered for combinatorial topology in [17]. The scheme in [17] does not involve private caches and it does not provide privacy against colluding users, whereas our model includes both. The comparison here is therefore not between the same problem settings, but rather at the level of memory usage for the same achievable rate R=Ct+r/Ct. Within this scope, the proposed scheme achieves lower total memory accessed by each user and global cache memory, while also ensuring a stronger privacy guarantee. Here, by total memory accessed by each user, we mean the total cache content available to each user. By global cache memory, we mean the total cache memory available across the entire system.

We first compare the total memory accessed by each user for the same rate in both schemes. The size of the total memory accessed by each user, $M_{1}$, for the SP-LFR scheme in [17], is given by

(39) M1=rNtC+rC−rtC−1r−1Ct.

The size of the total memory accessed by each user, $M_{2}$, for the proposed scheme is

(40) M2=rNtC+C−rtCt.

The rate *R* for both the schemes is Ct+rCt. By comparing (39) and (40), it is evident that, for the same rate, the total memory accessed by each user is lower in the proposed scheme.

**Figure 2:**
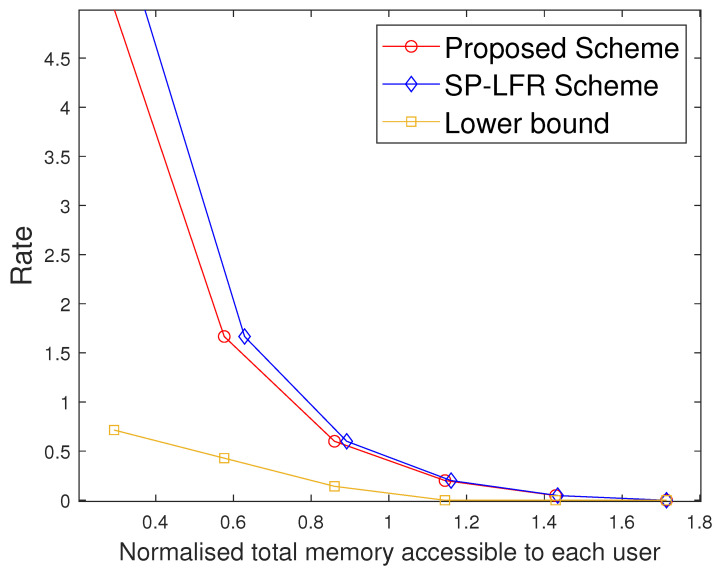
Performance comparison of our proposed scheme with the SP-LFR scheme in [17] when C=7,r=2,N=100.

**Figure 3:**
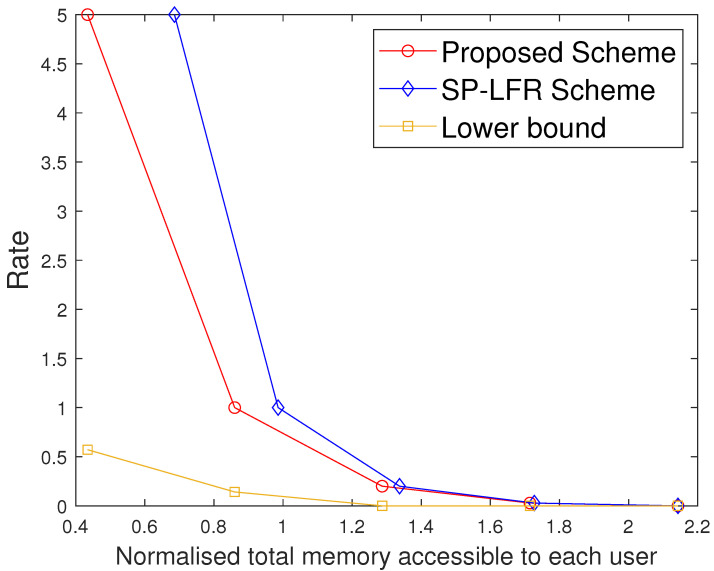
Performance comparison of our proposed scheme with the SP-LFR scheme in [17] when C=7,r=3,N=100.

**Figure 4:**
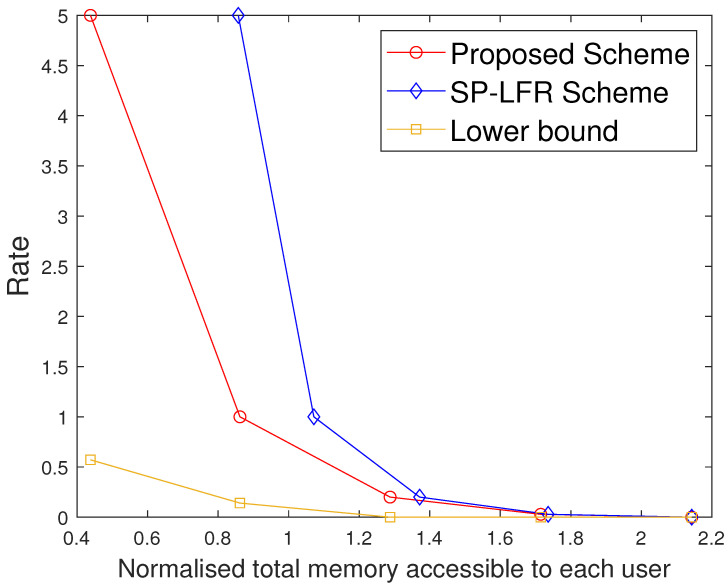
Performance comparison of our proposed scheme with the SP-LFR scheme in [17] when C=7,r=3,N=60.

Furthermore, we also compare the two schemes under the setting where the same total memory accessed by each user is considered for the two schemes. Suppose both schemes are operated at the same rate $R_{1}$, requiring total memory accessed by each user $M_{2}$ in the proposed scheme and $M_{1}$ in [17], with $M_{2}<M_{1}$. If we fix that to $M_{1}$ in both the systems, the proposed scheme has more memory available than strictly required for $R_{1}$, and hence can operate at a strictly smaller rate $R_{2}<R_{1}$, while the scheme in [17] remains at $R_{1}$. This behavior is also visible in Figure 2, Figure 3 and Figure 4, where for any fixed total memory accessed by each user, the red curve (proposed scheme) lies below the blue curve [17].

Next, we compare the global cache, which is the total memory available in the entire system. The global cache size, $M_{G}^{\left(1\right)}$, for the system in [17], is given by

(41) MG(1)=CNtC+CC−1r−1C−rtCt.

The global cache size, $M_{G}^{\left(2\right)}$, for the system considered in this work is

(42) MG(2)=CNtC+CrC−rtCt.

Since Cr<CC−1r−1, we have $M_{G}^{\left(2\right)}<M_{G}^{\left(1\right)}$. Thus, the global cache memory requirement is also lower for the system considered in this work.

**Figure 5:**
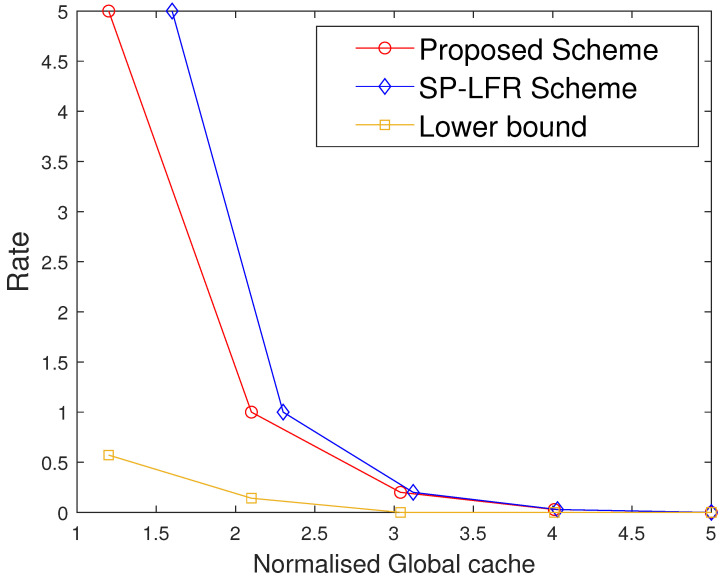
Performance comparison of our proposed scheme with the SP-LFR scheme in [17] considering the global cache when C=7,r=3,N=100.

**Figure 6:**
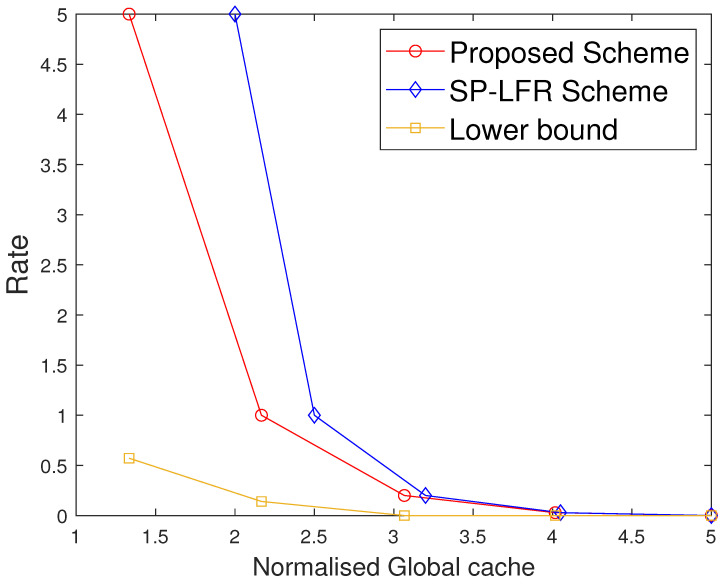
Performance comparison of our proposed scheme with the SP-LFR scheme in [17] considering the global cache when C=7,r=3,N=60.

Furthermore, we also compare the two schemes under the setting where the same global cache size is allocated. Suppose both schemes achieve the same rate $R_{1}$ with global cache sizes $M_{G}^{\left(2\right)}$ (proposed scheme) and $M_{G}^{\left(1\right)}$ [17], where $M_{G}^{\left(2\right)}<M_{G}^{\left(1\right)}$. If we fix the global cache size to $M_{G}^{\left(1\right)}$ in both systems, the proposed scheme has surplus memory available beyond what is necessary for $R_{1}$, enabling it to operate at a strictly smaller rate $R_{2}<R_{1}$, while the scheme in [17] remains at $R_{1}$. This advantage is consistent with Figure 5 and Figure 6, where for any fixed global cache size the red curve (proposed scheme) lies below the blue curve [17], confirming that our scheme achieves a lower rate under equal global cache memory.

**Theorem** **2.** *For the combinatorial topology with private caches,*

$$R^{*}\left(M_{M},M_{P}\right)\geq \max_{l\in \{1,2,,\min(N/2,K)\}}\frac{l\left\lfloor N/l\right\rfloor -(\min\left(l+r-1,C\right)M_{M}+lM_{P})}{\lfloor N/l\rfloor -1}.$$

**Proof.**  The detailed proof is given in Appendix B. □

**Theorem** **3.** For the combinatorial topology with private caches, the achievable rate R and the optimal rate $R^{*}$ satisfy

$$\frac{R(M_{M},M_{P})}{R^{*}\left(M_{M},M_{P}\right)}\leq \left\{\begin{array}{c} 1,\;r\geq C-1,N\geq 2K.\\ 5,\;\frac{CM_{M}}{N}=1,r<C-1,K\leq 5\left(r+1\right). \end{array}\right.$$

**Proof.**  The proof is presented in Appendix C. □

The scheme achieves optimality when r=C−1 because each user can access almost all caches, which maximizes the cached content available to each user and fully exploits coded multicasting opportunities. In this case, the achievable rate meets the converse bound (e.g., for r=C−1 and t=1, only one transmission is required). For smaller values of *r*, there remains a non-trivial gap. Since getting the gap results for any general *r* is mathematically rigorous, we provide an example to briefly explain the variation in the gap for different values of *r*.

**Example** **5.** *Consider C=7, N=100, and t=1.*

Table 1 reports the achievable rate R and the lower bound $R^{*}$ (Theorem 2) for different values of r. For r=1,2,…,5 there is a non-trivial gap between the two, while for r=C−1=6 we have $R=R^{*}$. This confirms that the proposed scheme is optimal at the threshold r=C−1, as stated in Theorem 3.

**Theorem** **4.** *Consider the GCT with private caches. For t∈[0:C], r∈[0:C], with $M_{M}=\frac{Nt}{C}$ and MP(r)=C−rtCt, there exists a scheme that satisfies the conditions in (26)–(28) with the rate, $R_{G}$ given by*

RG=∑r=0CKrCt+rCt.

**Proof.**  Consider the scheme given in Section 5. Each file is divided into Ct. So, the size of each subfile is 1/Ct. The total number of transmissions made are ∑r=0CKrCt+r. Each transmission has a size of 1/Ct. So, the rate is RG=∑r=0CKrCt+rCt. □

**Theorem** **5.** For the GCT with private cache, the optimal rate $R_{G}^{*}$ is given by,

RG*≥maxs∈[C]Ks(N−(sMM+∑r=0ssrKrMP(r)))N−1,

*where Ks=∑r=0ssrKr.*

**Proof.**  Refer Appendix D for the detailed proof. □

## 7. Discussion

In this section, we discuss the schemes presented in this paper through numerical evaluations. We compare the proposed scheme in Section 4 with the SP-LFR scheme in [17] and also with the MAN-PDA-based SP-LFR scheme in [15]. We also compare the performance of the scheme for GCT with private caches under different $K_{\mathrm{GCT}}$ vectors. The comparison is done in the following way:

- First, we compare the proposed scheme with the SP-LFR scheme in [17]. The system model in [17] is the multi-access combinatorial topology without private caches. However, the system model considered in this work is the combinatorial topology with private caches. To compare two different systems, we make use of two parameters. One is the total memory accessed per user, which is the total amount of memory available to each user. The other is the global cache size, which is the total amount of memory available in the entire system. Both systems support the same number of users for a given *r*.
- Next, we compare the proposed scheme with the scheme developed for the dedicated cache setup in [15]. The proposed scheme reduces to the dedicated cache setup in [15] when r=1. We first consider the case where both setups have the same number of caches. In the dedicated cache setup, the number of users is equal to the number of caches. However, in the combinatorial topology with private caches, the total number of users exceeds the number of caches. To enable a fair comparison under this setting, we use the metric of rate per user, defined as the rate divided by the total number of users. This metric was previously used in [3,7]. Next, we consider a comparison in which both the dedicated cache setup and the combinatorial topology with private caches serve the same number of users, and the total global cache size is also kept equal across both setups.
- Lastly, we compare the performance of the scheme for GCT with private caches for different values of the vector $K_{GCT}$ with the SP-LFR scheme in [17] and the MAN-PDA-based SP-LFR scheme in [15].

### 7.1. Comparision with the SP-LFR Scheme in [17]

The performance comparison between the proposed scheme and the SP-LFR scheme in [17] is given in Figure 2 for C=7,r=2,N=100,t∈[1:5]. Normalised total memory accessed per user, which is the total memory accessed by each user normalised with the total number of files, is used for comparison. The scheme in [17] does not include private caches and privacy against colluding users, while our model incorporates both. The comparison is therefore not between the same problem settings, but serves to illustrate the impact of introducing private caches. Since both schemes achieve the same rate expression, the curves can be meaningfully compared in terms of memory usage at equal rates. The proposed scheme performs better than the SP-LFR scheme in [17]. The performance comparison for C=7,r=3,N=100 is shown in Figure 3. The lower bound plotted in Figure 2 and Figure 3 corresponds to the information-theoretic lower bound derived in Theorem 2 using cut-set arguments. The gap between the proposed scheme and this lower bound is larger when the cache size is small because only limited coded multicasting opportunities exist in that regime. As the cache size grows, the multicasting gain increases, and the performance of the proposed scheme moves closer to the bound, which explains why the gap decreases. To achieve the rate of 5, the total memory accessed by each user in the SP-LFR scheme in [17] is 68.5714, whereas the proposed scheme requires 43.4286. The gap between the proposed scheme and the SP-LFR scheme in [17] increased when *r* increased from 2 to 3. This is because the keys that are required for C−1r−1 users are stored in each cache in the SP-LFR scheme in [17], which leads to an increase in the cache memory size. However, in the proposed scheme, due to the presence of a private cache, the keys that are useful to a particular user are stored in that user’s private cache. The performance comparison for C=7,r=3,N=60 is shown in Figure 4. As *N* increases, the gap between the curves decreases. The gap between the proposed scheme and the scheme in [17] increases with *r* and decreases with *N* because of the behavior of the second term in the total memory accessed per user expressions in (39) and (40). Specifically, both schemes share the same first term $\frac{rNt}{C}$, which represents the storage related to files, but differ in the key-storage term (second term). Our scheme stores each user’s keys exclusively in their private cache, whereas the scheme in [17] redundantly stores keys across the multi-access caches (to compensate for the absence of private caches). As *N* grows, the key-storage term becomes relatively less significant, so the gap shrinks. On the other hand, as *r* increases, the combinatorial factor in the second term grows, which increases the difference and hence increases the gap. The Normalised global cache(global cache/N) vs. rate curves are plotted in Figure 5 for C=7,r=3,N=100. The proposed scheme requires the global cache memory of 120 to achieve the rate of 5, whereas the SP-LFR scheme in [17] requires the global memory of 160. Similarly, the comparison curves for C=7,r=3,N=60 are shown in Figure 6. The performance gap widens as *r* increases and narrows as *N* increases. This can be explained by analyzing the global cache expressions in (41) and (42). The difference arises entirely from the key storage term (second term), since the file storage term Nt is identical. As *r* increases, the combinatorial factor CC−1r−1 grows faster than Cr. As *N* increases, the key storage term becomes negligible relative to Nt, causing the relative gap to shrink.

### 7.2. Comparision with the MAN-PDA Based SP-LFR Scheme in [15]

**Figure 7:**
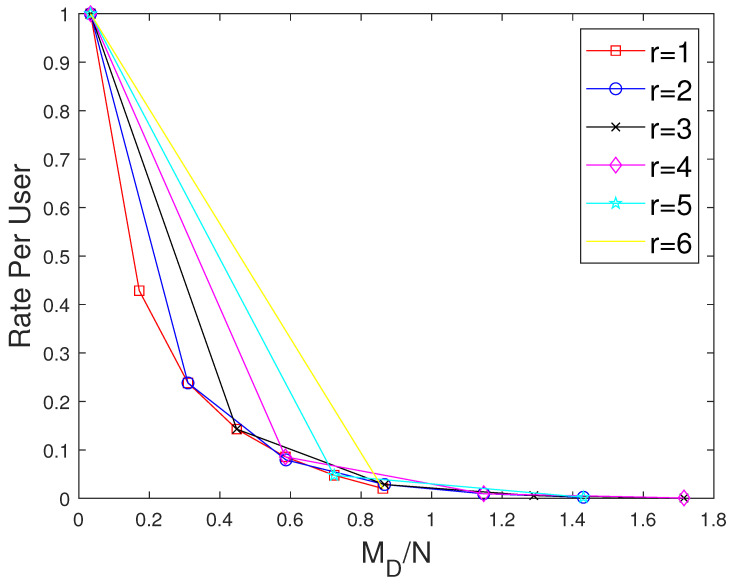
Performance comparison of our proposed scheme with [15] for different values of *r* considering the total memory accessed per user when C=7,N=30.

**Figure 8:**
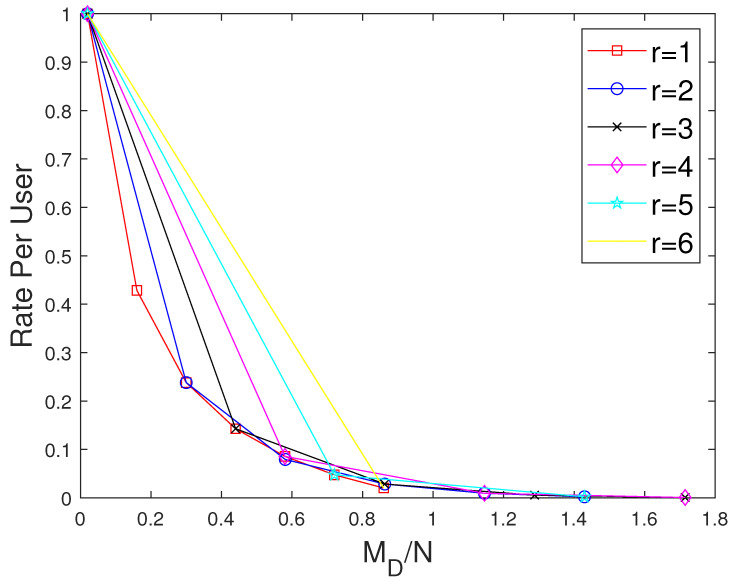
Performance comparison of our proposed scheme with [15] for different values of *r* considering the total memory accessed per user when C=7,N=50.

**Figure 9:**
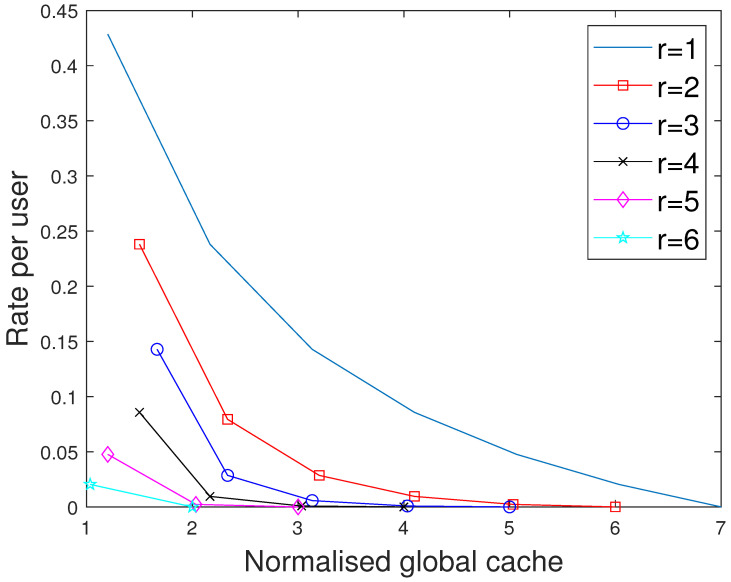
Performance comparison of our proposed scheme with [15] for different values of *r* considering the global cache when C=7,N=30.

**Figure 10:**
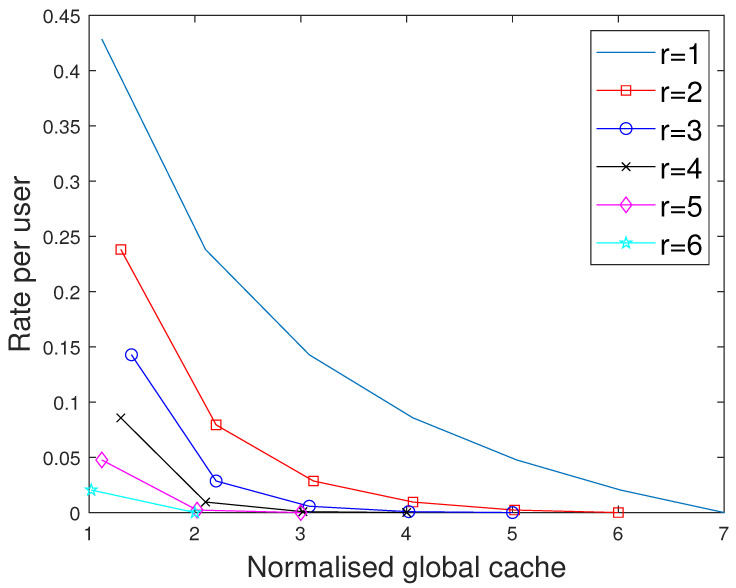
Performance comparison of our proposed scheme with [15] for different values of *r* considering the global cache when C=7,N=50.

**Figure 11:**
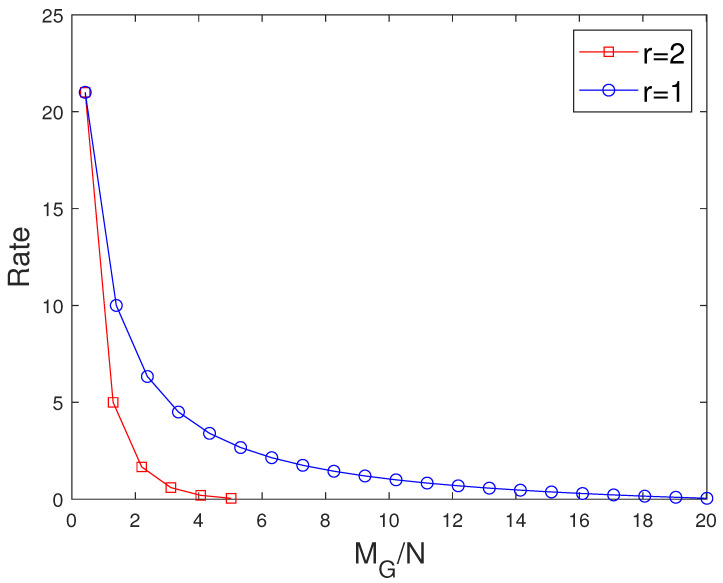
Performance comparison of our proposed scheme with [15] considering the same number of users when C=7,r=2,N=50.

**Figure 12:**
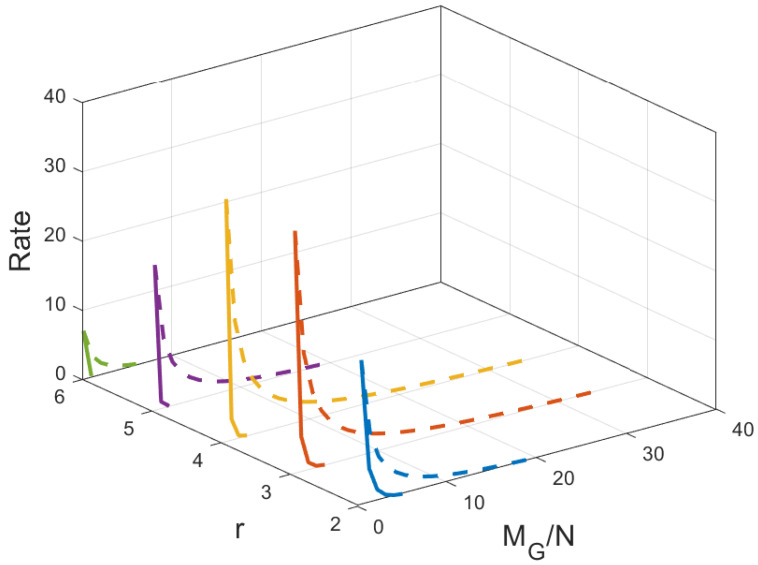
Performance comparison of our proposed scheme with [15] for different values of *r* considering the same number of users when C=7,N=50. The dotted lines represent the dedicated cache, and the solid lines represent the combinatorial topology with private caches.

The curves for r=1 show the performance of the MAN-PDA-based SP-LFR Scheme in [15]. As the number of users changes with *r*, to be able to compare the performances for different values of *r*, we use the rate per user instead of the rate. Let $M_{D}$ represent the size of cache memory in the dedicated cache setup. Now, we compare our scheme for different values of *r* when the total memory accessed by each user is the same. In the combinatorial topology with private caches, the total memory accessed by each user is $rM_{M}+M_{P}$. To make the users access the same amount of memory in both the setups, $M_{D}=rM_{M}+M_{P}$. The curves for different values of *r* are shown in Figure 7 when C=7,N=30 and in Figure 8 when C=7,N=50. The performance of the dedicated cache setup is better than our setup. This is because some subfiles are repeated in two or more caches that are accessible to a user, making the effective number of subfiles less in our setup when the total memory accessed by each user is the same. By this comparison, our setup is at a disadvantage because total system memory is more in the dedicated cache setup. For example, consider C=7,r=2,N=50. When $M_{M}=50/7$ and $M_{P}=5/7$, the global cache of the dedicated cache setup is 105, whereas for our setup, it is 65. Now, we compare the performance of curves for different values of *r*, keeping the global cache memory of the system the same. It is shown in Figure 9 and Figure 10 for N=30, and N=50, respectively. Clearly, as the value of *r* increases, the performance of the curves increases. So, the performance of our scheme is better than the scheme given in [15] for the dedicated cache setup. Now, we consider both the dedicated cache setup and the combinatorial topology with private caches setup to have the same number of users. Let the number of users in the dedicated cache setup be $K_{D}$. For a given *r*, the combinatorial topology with private cache setup supports Cr users. So, we make KD=Cr. For different values of *r*, we have to consider the dedicated cache setup with the number of users accordingly. Let $M_{G}$ represent the global cache memory. The performance curves are shown in Figure 11 for C=7,r=2. Clearly, the combinatorial topology with private caches outperforms the dedicated cache setup. The curves for all the values of *r* are shown in Figure 12, where the dotted lines represent the dedicated cache, and the solid lines represent the combinatorial topology with private caches. Figure 12 compares the performance of the proposed scheme for the combinatorial topology with private caches with the dedicated cache model from [15], under the condition that both systems serve the same number of users K=Cr. This is a crucial comparison because the combinatorial topology inherently supports K=Cr users—far more than the $K_{D}=C$ users supported by the dedicated cache setup. To enable a fair comparison, we scale the dedicated cache system to also serve KD=Cr users. The x-axis shows the normalized global cache size ($M_{G}/N$), and the y-axis shows the delivery rate *R*. For r=1, the two models coincide, while for r>1, the proposed scheme consistently outperforms the scheme in [15], achieving a strictly lower rate for the same $M_{G}/N$. For every r>1, our scheme achieves a strictly lower rate per user than the dedicated cache scheme. This clear shows that the proposed scheme outperfroms the scheme in [15].

### 7.3. Plots for GCT with Private Caches

**Figure 13:**
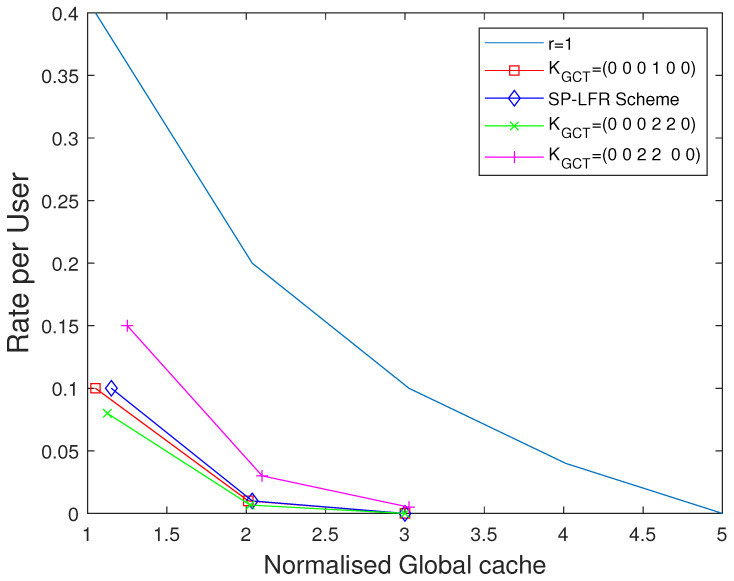
Performance comparison of our scheme for GCT with private caches with [15,17] considering different connectivities when C=5,N=80.

**Figure 14:**
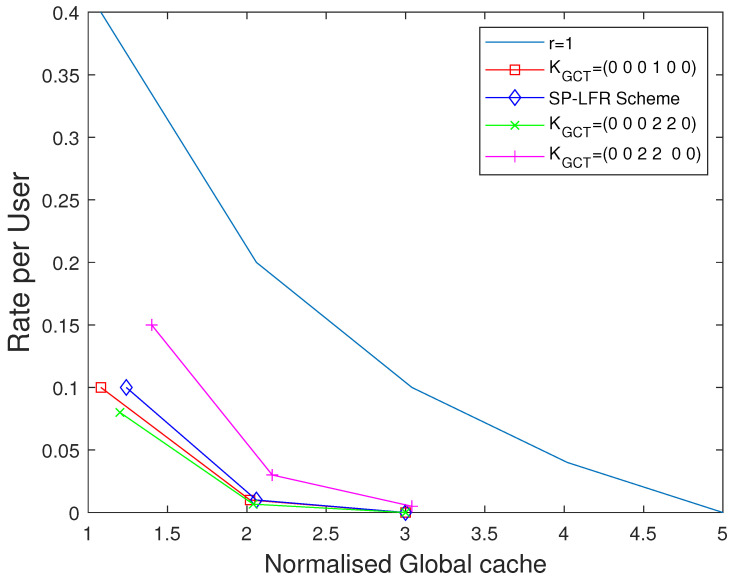
Performance comparison of our scheme for GCT with private cache with [15,17] considering different connectivities when C=5,N=50.

We now show the plots for the extended setup. Each user accesses a different number of multi-access caches in this setup, so we choose global cache memory as the metric. We choose to show the plots by considering different values for the vector $K_{GCT}$. When $K_{GCT}=\left(0,1,0,0,0,0\right)$, it represents the dedicated cache setup. The performance curves when $K_{GCT}=\left(0,0,0,1,0,0\right)$, $K_{GCT}=\left(0,0,2,2,0,0\right)$, and $K_{GCT}=\left(0,0,0,2,2,0\right)$ for N=50, and N=80 in Figure 13 and Figure 14, respectively. When t=0, the size of multi-access caches is zero. So, for the connectivities that serve more users, the global memory required is more to achieve the unity rate per user value. So, $K_{GCT}=\left(0,0,0,1,0,0\right)$ has less global cache than $K_{GCT}=\left(0,0,2,2,0,0\right)$, and $K_{GCT}=\left(0,0,0,2,2,0\right)$. For other values of *t*, the performance is shown in Figure 13 when N=80 and in Figure 14 when N=50. The curve shown as r=1 represents the dedicated cache setup. Clearly, the performance of the dedicated cache setup is lower when compared to the curves for GCT with private caches. The curve with $K_{GCT}=\left(0,0,0,2,2,0\right)$ is below the curve with $K_{GCT}=\left(0,0,0,1,0,0\right)$ because the contribution of the users that are connected to 4 multi-access caches makes the overall rate per user better. Similarly, the curve with $K_{GCT}=\left(0,0,2,2,0,0\right)$ is above the curve with $K_{GCT}=\left(0,0,0,1,0,0\right)$.

**Figure 15:**
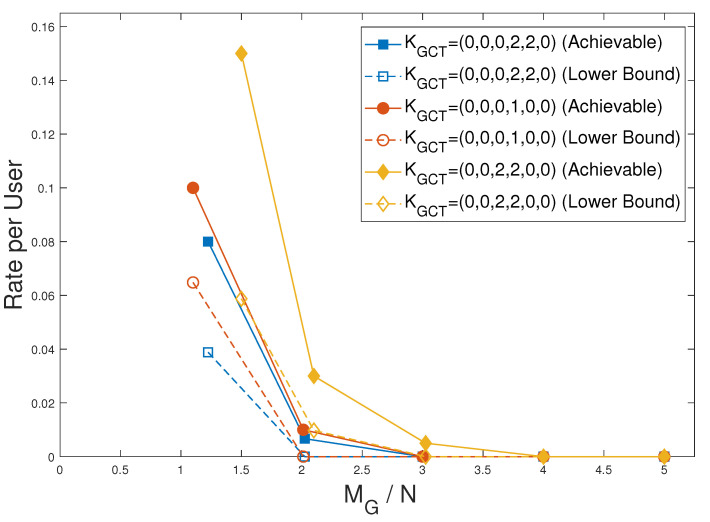
Plots showing the achievable scheme in Theorem 4 and the lower bound in Theorem 5 for multiple $K_{GCT}$ values.

We show the performance of the achievable scheme for GCT with private caches for multiple $K_{\mathrm{GCT}}$ values along with the lower bound given in Theorem 5 in Figure 15. It is evident that the gap between the achievable scheme and the lower bound is larger in the low-memory region, while it decreases as the global cache memory increases. The reason is that with more global cache memory, the number of coded multicasting opportunities grows, which in turn reduces the gap to the lower bound. This trend is shown in Table 2 for $K_{\mathrm{GCT}}=\left(0,0,2,2,0,0\right)$, C=5, N=80.

## 8. Conclusions

The security, demand privacy, and Linear Function Retrieval (LFR) for combinatorial topology with private caches are explored. We proposed a scheme that would satisfy the security, privacy, and LFR conditions. The private caches included in this work enable the proposed scheme to provide privacy against colluding users. It is shown that to achieve the same rate both in [17] and in the proposed scheme, the total memory accessed by each user is less in the proposed scheme. Our proposed scheme performs better than the SP-LFR scheme in [17] in terms of global cache memory also. As a special case, when r=1, the proposed scheme recovers the MAN-PDA-based SP-LFR scheme in [15]. We derived a lower bound and proved that the scheme is optimal when r≥C−1. Then, we showed that the proposed scheme can be extended to a more generalized setup.

## Figures and Tables

**Table 1:** Illustrative comparison for C=7, t=1, N=100.

*r*	K=Cr	$M_{M}$	$M_{P}$	*R*	Gap $R/R^{*}$
1	7	100/7≈14.2857	6/7≈0.8571	3.0	1.79
2	21	100/7≈14.2857	5/7≈0.7143	5.0	4.03
3	35	100/7≈14.2857	4/7≈0.5714	5.0	5.87
4	35	100/7≈14.2857	3/7≈0.4286	3.0	5.30
5	21	100/7≈14.2857	2/7≈0.2857	1.0	3.50
6	7	100/7≈14.2857	1/7≈0.1429	1/7≈0.1429	1.00

The Gap column shows the ratio $R/R^{*}$. At r=C−1=6, the scheme is optimal.

**Table 2:** Rate per user for $K_{\mathrm{GCT}}=\left(0,0,2,2,0,0\right)$, C=5, N=80.

*t*	Achievable	Lower Bound	Gap ($R/R^{*}$)	Observation
1	0.15	0.024	6.25	Large gap
2	0.03	0.005	6.0	Gap shrinking
3	0.005	0.001	5.0	Gap continues to shrink

## Data Availability

The original contributions presented in this study are included in the article. Further inquiries can be directed to the corresponding author.

## Appendix A. Proof of Proposition 1

We prove the minimum cache condition for security given in Proposition 1.

As shown in Figure 1, the server containing *N* files is connected to *K* users via an error-free shared link. Each user has access to a unique set of *r* caches from a total of *C* multi-access caches, each of size $M_{M}$, and also a private cache of size $M_{P}$. The condition is proved by considering single file retrieval, and the result holds for LFR as well. In this system, the total number of users is K=Cr. Since each user is associated with an *r*-sized subset, arrange all the users in the lexicographic order of their associated subsets. Let the $i^{th}$ user demand the file $W_{d_{i}}$. Let,

$$\bar{W}=\left\{W_{d_{1}},W_{d_{2}},\ldots ,W_{d_{K}}\right\},\;\bar{Z}=\left\{Z_{1}^{M},Z_{2}^{M},\ldots ,Z_{C}^{M}\right\}\cup \left\{Z_{1}^{P},\ldots ,Z_{K}^{P}\right\}.$$

By correctness condition in (8) and the security condition in (9), we can write

(A1) $$H(W_{d_{1}},W_{d_{2}},\ldots W_{d_{K}}|X,\bar{Z})=0,$$

(A2) $$I(W_{d_{1}},W_{d_{2}},\ldots W_{d_{K}};X)=0.$$

We have,

(A3a) CrF=H(W¯)

(A3b) $$\begin{array}{cc} \; & =I\left(\bar{W};X,\bar{Z}\right)+H\left(\bar{W}|X,\bar{Z}\right) \end{array}$$

(A3c) $$\begin{array}{cc} \; & =I(\bar{W};X,\bar{Z}) \end{array}$$

(A3d) $$\begin{array}{cc} \; & =I\left(\bar{W};X\right)+I\left(\bar{W};\bar{Z}|X\right) \end{array}$$

(A3e) $$\begin{array}{cc} \; & =I(\bar{W};\bar{Z}|X) \end{array}$$

(A3f) $$\begin{array}{cc} \; & \leq H(\bar{Z}) \end{array}$$

(A3g) $$\begin{array}{cc} \; & \leq \sum_{c=1}^{C}H\left(Z_{c}^{M}\right)+\sum_{k=1}^{K}H\left(Z_{k}^{P}\right) \end{array}$$

(A3h) $$\begin{array}{cc} \; & \leq CM_{M}F+KM_{P}F. \end{array}$$

This implies $CM_{M}+KM_{P}\geq K$. Here, (A3a) follows because there are Cr users, each demanding a file of size *F* bits, and the files are assumed independent and uniformly distributed; therefore the total entropy is CrF. Then, (A3b) follows from the definition of mutual information: for any two random variables *A* and *B*, H(A)=I(A;B)+H(A|B). Here, $A=\bar{W}$ and $B=(X,\bar{Z})$, so we obtain $H\left(\bar{W}\right)=I\left(\bar{W};X,\bar{Z}\right)+H\left(\bar{W}|X,\bar{Z}\right)$. Finally, (A3c) and (A3e) come from the correctness condition in (A1) and the security condition in (A2), respectively. This completes the proof of Proposition 1.

## Appendix B. Proof of Theorem 2

We prove the cut-set based lower bound given in Theorem 2.

Consider the combinatorial topology with private caches. Each user is connected to a unique set of *r* multi-access caches out of *C*, each of size $M_{M}$, and a private cache of size $M_{P}$. Since each user is associated with an *r*-sized subset, arrange all the users in the lexicographic order of their associated subsets. The content of a multi-access cache is represented as $Z_{i}^{M},i\in \left[C\right]$, and the content of a private cache is represented as $Z_{j}^{P},j\in \left[K\right]$. Each user is connected to *r* out of *C* multi-access caches and a private cache. So, if the first *l* users are considered, each user is connected to *r* out of z=min(l+r−1,C) multi-access caches and one private cache. The server consists of a library of *N* files, $W_{\left[N\right]}=\left\{W_{1},W_{2},\ldots W_{N}\right\}$, each of size *F* bits. Consider the first *l* users, let them request $W_{1},\ldots W_{l}$, respectively. The remaining users can request any arbitrary files. We consider that each user requests a single file. The lower bound is also valid for linear function retrieval. Let the server make the transmission $X_{1}$. Using the transmission $X_{1}$, the content of the first z=min(l+r−1,C) multi-access caches, and *l* private caches, the first *l* users can decode $W_{1},\ldots W_{l}$. Now, let the first *l* users request $W_{l+1},W_{l+2},\ldots W_{2l}$, respectively. The server sends the transmission $X_{2}$. Using the transmission $X_{2}$, the first *z* multi-access caches, and the *l* private caches, the first *l* users can decode the files $W_{l+1},W_{l+2},\ldots W_{2l}$. By continuing in this way, consider the transmission $X_{\left\lfloor N/l\right\rfloor }$. The first *l* users can decode $W_{(\lfloor N/l\rfloor -1)l+1},W_{(\lfloor N/l\rfloor -1)l+2},\ldots W_{(\lfloor N/l\rfloor )l}$ files using the first *z* mutli-access caches and the first *l* private caches. Considering all the transmissions $X_{1},X_{2},\ldots X_{\left\lfloor N/l\right\rfloor }$, using the first *z* multi-access caches and the first *l* private caches, the users must be able to decode $W_{1},W_{2},\ldots W_{l\lfloor N/l\rfloor }$. Let,

$$\begin{array}{cccc} \tilde{W}= & \{W_{1},W_{2},\ldots ,W_{l\lfloor N/l\rfloor }\}, & \tilde{X}= & \{X_{1},X_{2},\ldots ,X_{\left\lfloor N/l\right\rfloor }\},\\ \tilde{X}/s= & \{X_{1},\ldots ,X_{s-1},X_{s+1},\ldots ,X_{\left\lfloor N/l\right\rfloor }\}, & \tilde{Z}= & \left\{Z_{1}^{M},\ldots ,Z_{z}^{M}\right\}\cup \left\{Z_{1}^{P},\ldots ,Z_{l}^{P}\right\} \end{array}$$

By the conditions in (8) and (9),

(A4) $$H(\tilde{W}|\tilde{X},\tilde{Z})=0,$$

(A5) $$I\left(\tilde{W};X_{s}\right)=0,\;s=1,2\ldots \left\lfloor N/l\right\rfloor .$$

By using the transmissions in $\tilde{X}$, the first *l* users can decode the files in $\tilde{W}$ using the cache content in $\tilde{Z}$. We have,

(A6a) $$\begin{array}{cc} l\lfloor N/l\rfloor F & =H(\tilde{W}) \end{array}$$

(A6b) $$\begin{array}{cc} \; & =I\left(\tilde{W};\tilde{X},\tilde{Z}\right)+H\left(\tilde{W}|\tilde{X},\tilde{Z}\right) \end{array}$$

(A6c) $$\begin{array}{cc} \; & =I(\tilde{W};\tilde{X},\tilde{Z}) \end{array}$$

(A6d) $$\begin{array}{cc} \; & =I(\tilde{W};\left\{X_{1},X_{2},\ldots X_{\left\lfloor N/l\right\rfloor }\right\},\tilde{Z}) \end{array}$$

(A6e) $$\begin{array}{cc} \; & =I\left(\tilde{W};X_{s}\right)+I\left(\tilde{W};\tilde{X}/s,\tilde{Z}/X_{s}\right) \end{array}$$

(A6f) $$\begin{array}{cc} \; & =I(\tilde{W};\tilde{X}/s,\tilde{Z}/X_{s}) \end{array}$$

(A6g) $$\begin{array}{cc} \; & \leq H(\tilde{X}/s,\tilde{Z}) \end{array}$$

(A6h) $$\begin{array}{cc} \; & \leq \sum_{i=1,i\neq s}^{\left\lfloor N/l\right\rfloor }H\left(X_{i}\right)+H\left(\tilde{Z}\right) \end{array}$$

(A6i) $$\begin{array}{cc} \; & \leq \left(\left\lfloor N/l\right\rfloor -1\right)R^{*}F+zM_{M}F+lM_{P}F. \end{array}$$

where (A6a) follows because the set $\tilde{W}$ contains ⌊N/l⌋l independent files of size *F* each, giving total entropy $H\left(\tilde{W}\right)=\left\lfloor N/l\right\rfloor \;l\;F$, and (A6b) follows from the definition of mutual information: For any random variables *A* and *B*, H(A)=I(A;B)+H(A∣B). Here, $A=\tilde{W}$ and $B=(X,\tilde{Z})$, where *X* denotes the transmissions and $\tilde{Z}$ denotes the relevant cache contents. Now, (A6c) comes from the condition in (A4) and (A6f) comes from the security condition in (A5). By rearranging the terms, we get

$$R^{*}\geq \frac{l\left\lfloor N/l\right\rfloor -\left(zM_{M}+lM_{P}\right)}{\lfloor N/l\rfloor -1}.$$

Optimizing over all possible values of *l*, we obtain

$$R^{*}\geq \max_{l\in \{1,2,\ldots \min(N/2,K)\}}\frac{l\left\lfloor N/l\right\rfloor -\left(zM_{M}+lM_{P}\right)}{\lfloor N/l\rfloor -1}.$$

This completes the proof.

## Appendix C. Proof of Theorem 3

Consider the combinatorial topology with private caches. The server has *N* files, each of size *F* bits and it is connected to *K* users through an error-free shared link. Each user has access to a unique set of *r* out of *C* mutli-access caches and a private cache. For every set of *r* mutli-access caches, there is a user. The rate of the scheme with $M_{M}=Nt/C$ and MP=C−rt/Ct that satisfies the conditions in (8)–(10) is given by

(A7) R(MP,MM)=Ct+rCt.

The optimal rate $R^{*}$ is given by

(A8) $$R^{*}\geq \max_{l\in \{1,2,\ldots \min(N/2,K)\}}\frac{l\left\lfloor N/l\right\rfloor -\left(zM_{M}+lM_{P}\right)}{\lfloor N/l\rfloor -1},$$

where z=min(l+r−1,C). Now, we prove the optimality results using the inequality in (A8). First, we consider the case when r≥C−1. When r=C−1, the possible values for *t* are t=0,1. Let us consider the case when t=0. The multi-access cache size $M_{M}=\frac{Nt}{C}=0$ and private cache size $M_{P}=1$. Now, consider Equation (A8) for l=K. Then, z=C. The optimal rate for l=K in (A8) is

(A9a) $$\begin{array}{cc} R^{*} & \geq \frac{K\left\lfloor N/K\right\rfloor -\left(CM_{M}+KM_{P}\right)}{\lfloor N/K\rfloor -1} \end{array}$$

(A9b) $$\begin{array}{cc} \; & =\frac{K\lfloor N/K\rfloor -K}{\lfloor N/K\rfloor -1} \end{array}$$

(A9c) $$\begin{array}{cc} \; & =\frac{K(\lfloor N/K\rfloor -1)}{\lfloor N/K\rfloor -1} \end{array}$$

(A9d) $$\begin{array}{cc} \; & =K, \end{array}$$

where (A9a) comes by substituting $M_{M}=0$ and $M_{P}=1$.

For t=0, the achievable rate given (A7) is

(A10) R(0,1)=K.

Thus, it is proved for t=0. Now consider the case when t=1,r=C−1. The multi-access memory size $M_{M}$ and the private cache memory size $M_{P}$ is

MM=NtC=NC,MP=C−rtCt=1C.

Let us calculate the rate. By substituting the values of *t* and *r* in (A7), we get

R(NC,1C)=C1+C−1C=1C.

Now, let us calculate $M=rM_{M}+M_{P}$ as follows

$$\begin{array}{cc} M & =rM_{M}+M_{P}=\frac{Nr}{C}+\frac{1}{C}\\ \; & =\frac{N(C-1)}{C}+\frac{1}{C}=N\left(1-R\right)+R. \end{array}$$

By rearranging the above terms, we get

(A11) $$R=\frac{N-M}{N-1}.$$

Now, consider the optimal rate equation in (A7) when l=1 and substitute the values of $M_{M}$ and $M_{P}$.

$$\begin{array}{cc} R^{*} & \geq \frac{N-(rM_{M}+M_{P})}{N-1}\\ \; & =\frac{N-M}{N-1}. \end{array}$$

By comparing the above inequality with (A11), the proof for t=1 is completed for r=C−1. Suppose r=C, then the only possible value of *t* is t=0. The achievable rate and the optimal rate for t=0 is given in (A10) and (A9d), respectively, which proves the optimality.

Now, let us consider the case when t=1,r<C−1. The values of $M_{M}$ and $M_{P}$ for t=1 are as given below.

$$\begin{array}{cc} M_{M} & =\frac{N}{C},\\ M_{P} & =\frac{C-r}{C}. \end{array}$$

Now, consider the optimal rate inequality in (A7) when l=1 and substitute the values of $M_{M}$ and $M_{P}$.

$$\begin{array}{cc} R^{*} & \geq \frac{N-(rM_{M}+M_{P})}{N-1}\\ \; & =\frac{N-(\frac{Nr}{C}+\frac{C-r}{C})}{N-1}\\ \; & =\frac{\left(\frac{N(C-r)}{C}-\frac{C-r}{C}\right)}{N-1}\\ \; & =\frac{\left(1-\frac{r}{C}\right)\left(N-1\right)}{N-1}\\ \; & =1-\frac{r}{C}. \end{array}$$

The rate, *R* given in (A8) for t=1 is given by

R=Cr+1C.

The above equation can be simplified as follows.

R=Cr+1C=C!(C−r−1)!(r+1)!C=(C−r)Cr(r+1)C=(1−rC)(Crr+1).

Therefore at t=1,

RR*≤(1−rC)(Crr+1)1−rC≤Crr+1≤5,ifK≤5(r+1).

This completes the proof.

## Appendix D. Proof of Theorem 5

We prove the cut-set based lower bound for GCT with private cache given in Theorem 5.

We consider GCT with private caches. There are *C* multi-access caches and *K* private caches in the system. Assume that the users are arranged as follows: The users that are connected to *r* multi-access caches are followed by the users that are connected to r+1 multi-access caches. The users that are connected to the same number of multi-access caches are arranged in lexicographic order. Consider the first *s* multi-access caches where s∈[1:C]. The number of users who access only from these *s* caches is Ks=∑i=0ssiKi. Let those $K^{s}$ users demand $W_{1},\ldots W_{K^{s}}$. The remaining users demand arbitrary files from $W_{\left[N\right]}$. Let the server send the transmission $X_{1}$ to satisfy the demands of the users. Using the transmission $X_{1}$, the first *s* multi-access caches, and $K^{s}$ private caches, the considered $K^{s}$ users can decode the first $K_{s}$ files. Let ${\tilde{Z}}_{i}$ denote the total cache content accessible to the $i^{th}$ user. Now, consider the case where the $K^{s}$ users request $W_{K^{s}+1},W_{K^{s}+2},\ldots W_{2K^{s}}$. Let the server send $X_{2}$ to satisfy the demands. Using the transmission $X_{2}$, the first *s* multi-access caches, and $K^{s}$ private caches, the considered $K^{s}$ users can decode the $W_{K^{s}+1},W_{K^{s}+2},\ldots W_{2K^{s}}$ files. If we consider $L=\lceil N/K^{s}\rceil$ such demand vectors and the corresponding transmissions, then all the *N* files can be decoded by the $K^{s}$ users. The decodability and security conditions are as follows:

(A12) $$H(W_{\left[N\right]}|X_{\left[L\right]},{\tilde{Z}}_{\left[K^{s}\right]})=0.$$

(A13) $$I(W_{\left[N\right]};X_{L})=0.$$

We have,

(A14a) $$\begin{array}{cc} N & =H(W_{\left[N\right]} \end{array}$$

(A14b) $$\begin{array}{cc} \; & =I\left(W_{\left[N\right]};X_{\left[L\right]},{\tilde{Z}}_{\left[K^{s}\right]}\right)+H\left(W_{\left[N\right]}|X_{\left[L\right]},{\tilde{Z}}_{\left[K^{s}\right]}\right) \end{array}$$

(A14c) $$\begin{array}{cc} \; & =I(W_{\left[N\right]};X_{\left[L\right]},{\tilde{Z}}_{\left[K^{s}\right]}) \end{array}$$

(A14d) $$\begin{array}{cc} \; & =I\left(W_{\left[N\right]};X_{L}\right)+I\left(W_{\left[N\right]};X_{\left[L-1\right]},{\tilde{Z}}_{\left[K^{s}\right]}/X_{L}\right) \end{array}$$

(A14e) $$\begin{array}{cc} \; & =I(W_{\left[N\right]};X_{\left[L-1\right]},{\tilde{Z}}_{\left[K^{s}\right]}/X_{L}) \end{array}$$

(A14f) $$\begin{array}{cc} \; & \leq \sum_{l=1}^{L-1}H\left(X_{l}\right)+\sum_{j=1}^{K^{s}}H\left({\tilde{Z}}_{K^{s}}\right) \end{array}$$

(A14g) ≤(L−1)R*+sMM+∑r=0ssrKrMP(r),

where (A14a) comes because the files are independently and uniformly distributed, and (A14b) come from the definition of mutual information. Then, (A14c) and (A14e) comes from the conditions in (A12) and (A13), respectively. As $\left\lceil \frac{N}{K^{s}}\right\rceil \leq \frac{N}{K^{s}}+\frac{K^{s}-1}{K^{s}}$.

(A15a) N≤(NKs+Ks−1Ks−1)RG*+sMM+∑r=0ssrKrMP(r),

(A15b) =(N−1Ks)RG*+sMM+∑r=0ssrKrMP(r).

By rearranging the terms we get,

RG*≥Ks(N−(sMM+∑r=0ssrKrMP(r)))N−1.

By maximizing over all possible values of s∈[1:C], we get

R*≥maxs∈[C]Ks(N−(sMM+∑r=0s.srKrMP(r)))N−1.

This completes the proof.
